# Replication timing and epigenome remodelling are associated with the nature of chromosomal rearrangements in cancer

**DOI:** 10.1038/s41467-019-08302-1

**Published:** 2019-01-24

**Authors:** Qian Du, Saul A. Bert, Nicola J. Armstrong, C. Elizabeth Caldon, Jenny Z. Song, Shalima S. Nair, Cathryn M. Gould, Phuc-Loi Luu, Timothy Peters, Amanda Khoury, Wenjia Qu, Elena Zotenko, Clare Stirzaker, Susan J. Clark

**Affiliations:** 1 Epigenetics Laboratory, Genomics and Epigenetics Division, Garvan Institute of Medical Research, Sydney, 2010 NSW Australia; 2 St. Vincent’s Clinical School, Faculty of Medicine, UNSW Sydney, Sydney, 2010 NSW Australia; 3 Mathematics and Statistics, School of Engineering and Information Technology, Murdoch University, Perth, 6150 WA Australia; 4 Replication and Genome Stability, Cancer Division, Garvan Institute of Medical Research, Sydney, 2010 NSW Australia

## Abstract

DNA replication timing is known to facilitate the establishment of the epigenome, however, the intimate connection between replication timing and changes to the genome and epigenome in cancer remain largely uncharacterised. Here, we perform Repli-Seq and integrated epigenome analyses and demonstrate that genomic regions that undergo long-range epigenetic deregulation in prostate cancer also show concordant differences in replication timing. A subset of altered replication timing domains are conserved across cancers from different tissue origins. Notably, late-replicating regions in cancer cells display a loss of DNA methylation, and a switch in heterochromatin features from H3K9me3-marked constitutive to H3K27me3-marked facultative heterochromatin. Finally, analysis of 214 prostate and 35 breast cancer genomes reveal that late-replicating regions are prone to *cis* and early-replication to *trans* chromosomal rearrangements. Together, our data suggests that the nature of chromosomal rearrangement in cancer is related to the spatial and temporal positioning and altered epigenetic states of early-replicating compared to late-replicating loci.

## Abstract (web summary)

The connection between DNA replication timing and changes that occur to the epigenome in cancer are still poorly understood. Here, the authors perform Repli-Seq and integrated epigenome analyses and find that genomic regions that undergo long-range epigenetic deregulation in prostate cancer also show concordant differences in replication timing.

## Introduction

Replication of the mammalian genome is an essential process that guarantees the accurate copying of genetic information before cell division. Each round of replication represents an opportunity for error, leading to the acquisition of mutations^1^ and copy number aberrations^2–4^. Epigenetic maintenance factors are also associated with the DNA replication machinery^5^ and therefore DNA replication represents a similar opportunity for deregulation of the epigenome. The DNA replication timing program of the cell is highly organised and defined as the temporal sequence of locus replication events that occur during the synthesis phase (S-phase) of the cell cycle, from early to late^6,7^. Replication timing has been shown to stratify many features of the genome and epigenome, including gene density, gene transcription, histone modifications, DNA methylation and three-dimensional (3D) chromatin organisation^8–13^. Generally, active and open euchromatin regions are replicated early in S-phase, and repressed and closed heterochromatin regions are replicated late in S-phase^7^. Studies of mouse embryonic stem cell differentiation show that re-organisation of the replication timing program is accompanied by a concomitant re-organisation of the epigenome across large domains^14,15^. As the replication timing program contributes to both epigenetic maintenance and cell identity, disruption of these processes could be a key cellular event that also contributes to carcinogenesis. However, the relationship between replication timing and epigenome alterations in cancer, and the combined impact on shaping the genomic landscape of tumour cells has remained largely unexplored.

We and others have previously shown that epigenetic deregulation in cancer can span large domains of long-range epigenetic silencing (LRES) and activation (LREA) with coordinated gene expression, histone modification, DNA methylation changes and disruption of topologically associated domains (TADs) over several kilobases to megabases^16–18^. Ryba et al. (2012) also reported that up to 18% of the genome can change in replication timing in acute lymphoblastic leukaemia^19^. Therefore, given the long-range domain level of epigenetic change observed in cancer, we were motivated to ask what is the relationship between replication timing and associated alterations to the epigenome and genome in cancer.

Here, we use high-resolution epigenome and genome-wide characterisation of normal and cancer cells to investigate how the replication timing landscape is associated with the cancer-specific epigenome changes and chromosomal rearrangements observed in prostate and breast cancers. We find that the differences in epigenetic deregulation between early and late replication underpin long-range epigenetic deregulation and potentially shape the nature of cancer mutational landscape.

## Results

### Replication timing is largely conserved in cancer cells

**Fig. 1:**
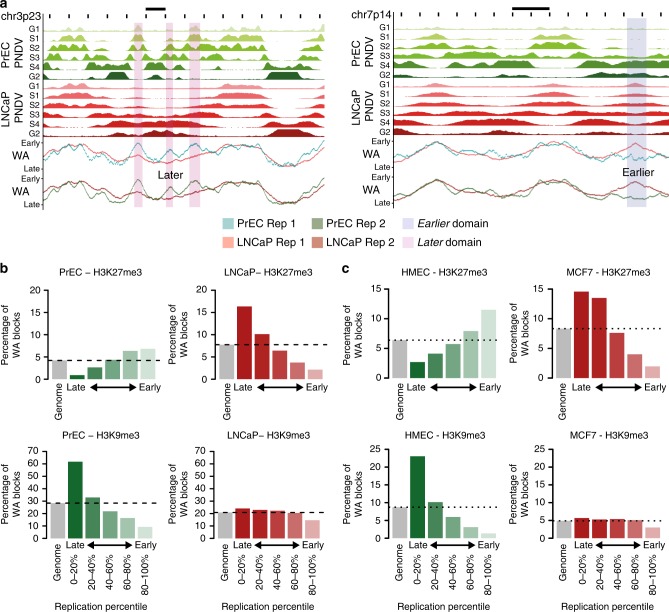
Relationship between chromatin and replication timing in normal and cancer. **a** Representative examples of regions of timing change between PrEC and LNCaP. PNDVs for PrEC (green) and LNCaP (red) are shown in the upper panels. The summarised WA values for each replicate are beneath. Regions that replicate *later* in LNCaP (ΔWA < −25) are highlighted in pink. Regions that replicate *earlier* in LNCaP (ΔWA > 25) are highlighted in blue. Scale bars represent 1 Mb. **b** Percentage occupancy of chromatin marks for 1 kb loci (WA blocks) within replication timing percentiles for repressive marks H3K27me3 and H3K9me3 in PrEC and LNCaP. **c** Percentage occupancy of chromatin marks for 1 kb loci (WA blocks) within replication timing percentiles for repressive marks H3K27me3 and H3K9me3 in HMEC and MCF7

To determine if there are changes in replication timing in normal and cancer prostate cells, we performed Repli-Seq^6,20^ in duplicate in normal prostate cells (PrEC) and prostate cancer (LNCaP) cells (see Methods, Supplementary Figure 1a–f). To examine the nature of the replication timing landscape, we plotted the replication timing weighted average (WA) values for all ~2.8 million mappable 1 kb bins (‘loci’) (Supplementary Figure 1g) and found that 94.3% of loci are remarkably conserved in the cancer cells, using a stringent WA difference of 25 (|ΔWA| < 25) (Supplementary Figure 1h). In fact, only 5.7% of the genome showed a difference in replication timing; 3.2% of the genome replicated *later* in LNCaP compared to PrEC (ΔWA < −25), and 2.5% replicated *earlier* in LNCaP compared to PrEC (ΔWA > 25) (Supplementary Figure 1g). To identify domains of consecutive loci where the time of replication is altered, we merged all loci within 50 kb that had a |ΔWA| > 25, and found 314 domains replicated *later* and 244 domains replicated *earlier* in LNCaP compared to PrEC. The *later* and *earlier* domains are distributed across the genome spanning all chromosomes (visualised in Supplementary Figure 2). Exemplary domains that replicate *later* and *earlier* in the cancer cells are also shown in Fig. 1a.

### Differences in heterochromatin occur in late-replication

To next investigate where replication timing stratifies the epigenome and transcriptome, we examined gene expression and chromatin differences between PrEC and LNCaP at early- and at late-replicating loci. Similar to previous reports in other cell types^6,11^, both prostate normal and cancer cells display high gene density and high expression in early-replicating loci, and low gene density and constitutively low gene expression in late-replicating loci (Supplementary Figure 3a, b). The prostate cells also display positive associations^6,21^ between early-replication and active chromatin marks, and late-replication and repressive chromatin marks (Supplementary Figure 3c). Moreover, we find that the distribution of active and permissive marks, including H3K4me3, H3K27ac, H3K4me1, H2AZac and H3K36me3 and DNAse1 hypersensitivity (HS), are progressively enriched towards early-replicating loci, whereas the repressive marks, H3K9me3, lamin A/C and lamin B1, are progressively enriched towards late-replicating loci (Fig. 1b, Supplementary Figure 4a, b). Similar chromatin and replication timing associations were identified in normal breast epithelial cell line (HMEC) and breast cancer cell line (MCF7) (Fig. 1c, Supplementary Figure 3d, Supplementary Figure 4c).

Notably, only two of the chromatin marks, H3K27me3 and H3K9me3, show different distributions across replication timing between the normal and cancer cells. First, H3K27me3 is more enriched in early-replicating loci than in late-replicating loci in PrEC, whereas in LNCaP cells, H3K27me3 is more enriched in late-replicating loci than early (Fig. 1b). Second, H3K9me3 is enriched in late-replicating loci compared to early-replicating loci in normal PrEC, whereas, in the cancer cells H3K9me3 enrichment is substantially reduced in late timing (Fig. 1b). Opposing H3K27me3 and H3K9me3 enrichment at late-replicating loci is also seen between HMEC and MCF7 (Fig. 1c). Both H3K27me3 and H3K9me3 remodelling has been reported to occur in cancer^22,23^, but an inverse relationship has not previously been associated with replication timing.

Next, we investigated whether the chromatin state is also altered in cancer cells within the regions that *change* in replication timing. Generally, we find that LNCaP cells show a gain of permissive marks at *earlier* replicating loci and loss of permissive marks at *later* replicating loci (Supplementary Figure 4d). Conversely, we find a loss of repressive marks in *earlier* replicating loci and a gain of repressive marks in *later* replicating loci (Supplementary Figure 4e). Interestingly, H3K27me3 does not show the same trend of reciprocal association as other repressive marks, but in contrast only shows enrichment towards *later* loci in LNCaP compared to PrEC (Supplementary Figure 4e). Therefore, genome-wide remodelling of H3K27me3 appears to specifically occur at loci that replicate *later* in cancer cells as well as regions of constitutive late-replication.

### Replication timing states stratify methylation alterations

**Fig. 2:**
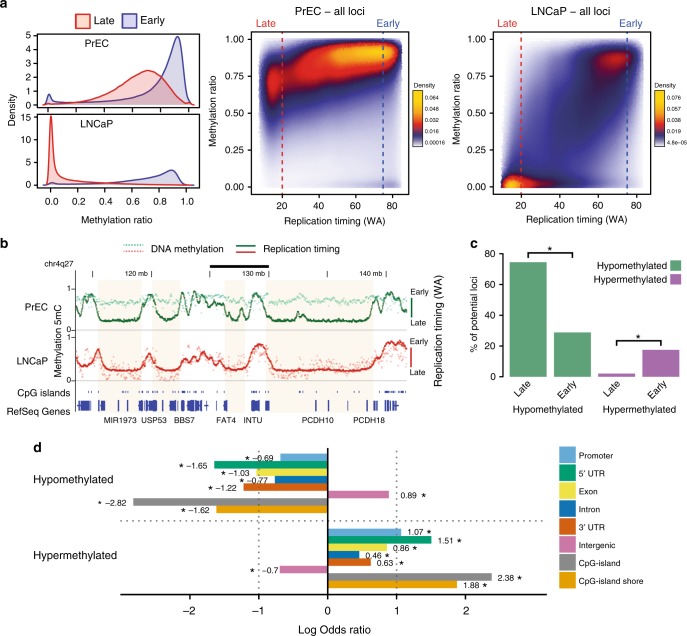
Replication timing correlates with DNA methylation in cancer. **a** DNA methylation (WGBS) density distributions for early (blue) and late (red) loci per cell line. Adjacent are scatterplots of DNA methylation in relation to replication timing (WA) for all measured 1 kb loci in PrEC and LNCaP. Blue dashed line indicates early (WA > 75) and red dashed line indicates late (WA < 20). **b** Representative examples of late-replicating regions common to both PrEC and LNCaP (shaded) that become hypomethylated in LNCaP. The correlation between DNA methylation and replication timing values increases from PrEC (Spearman’s 0.3073, *p* < 2.2e−16) to LNCaP (Spearman’s 0.4985, *p* < 2.2e−16). Scale bar represents 5 Mb. **c** Percentage (*y*-axis) of PrEC early and late regions (1 kb loci) that are hypermethylated (LNCaP-PrEC, ΔWGBS > 0.2) and hypomethylated (LNCaP-PrEC, ΔWGBS < −0.2). Asterisks indicate significantly different percentages between early and late (test of equal proportions, *p* < 2.2e−16). **d** Associations between genomic elements and hypo- and hypermethylated 1 kb loci. Association is above zero, and disassociation is below zero. Asterisks indicate significant associations (FDR < 0.05, Fisher’s exact test)

To investigate the relationship between replication timing and DNA methylation, we performed whole genome bisulfite sequencing (WGBS) in prostate and breast normal and cancer cell lines and clinical samples. We find that for normal prostate and breast cells (PrEC and HMEC), late-replicating loci are less methylated compared to early-replicating loci (Fig. 2a, Supplementary Figure 5a), similar to other cell types^12,13,24^. Notably, there is an even greater loss of DNA methylation at late-replicating loci in cancer cells (LNCaP and MCF7) relative to normal cells (Fig. 2a, Supplementary Figure 5a). Furthermore, we observe similar trends in the preferential loss of methylation at late-replication loci relative to early loci in clinical prostate and breast cancer WGBS datasets (Supplementary Figure 5b, c).

Moreover on a broad scale, methylation is reduced in LNCaP compared to PrEC at regions of constitutive late replication (Fig. 2b, Supplementary Figure 6a). However, unlike in PrEC where the DNA methylation and replication timing profiles are largely distinct, the genome-wide DNA methylation profile is more in synchrony with the replication timing profile in LNCaP cells (Fig. 2b, Supplementary Figure 6a). Furthermore, we find more hypomethylated (LNCaP − PrEC, ΔmCG < −0.2) than hypermethylated (LNCaP − PrEC, ΔmCG > 0.2) loci genome-wide (Fig. 2c), and hypomethylation is significantly more enriched in late-replicating loci compared to early-replicating loci (74% vs. 28%, test of equal proportions *p* < 2.2e−16). Conversely, hypermethylation is significantly enriched in early-replicating loci compared to late-replicating loci (17% vs. 2%, test of equal proportions *p* < 2.2e−16) (Fig. 2c). Notably, hypomethylated loci in cancer cells are primarily enriched for intergenic regions, whereas hypermethylated loci are enriched for CpG-islands, CpG-island shores and promoter/5′ UTR regions (Fig. 2d). We further observe the association of partially methylated domains (PMDs) and lamina-associated domains (LADs) with the hypomethylated loci, and disassociation with hypermethylated loci (Supplementary Figure 6b). Together, the data shows that cancer-associated DNA hypermethylation occurs preferentially in early-replication at CpG-islands and hypomethylation in late-replication at intergenic regions.

### Alterations in replication timing modulate gene expression

**Fig. 3:**
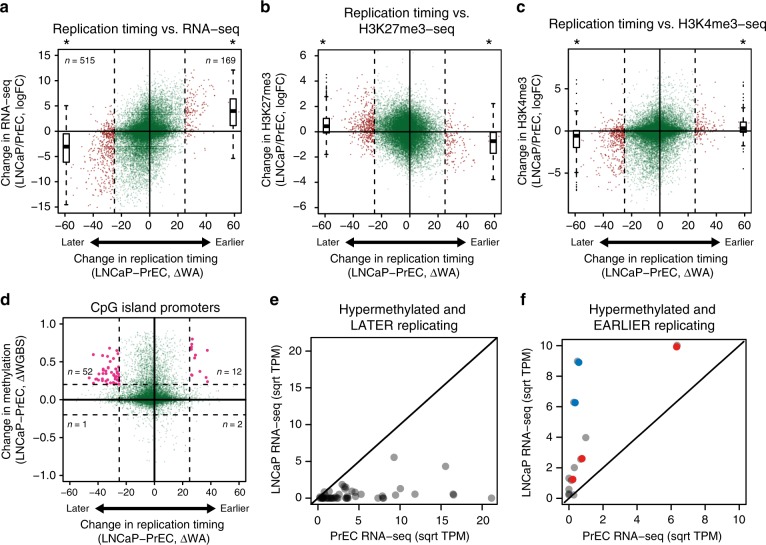
Relationship between replication timing, DNA methylation and expression. **a** Difference in replication timing (ΔWA) compared to the change in gene expression (log fold change, logFC). **b**, **c** Scatterplots showing the relationship between change in replication timing and change in H3K27me3 and H3K4me3 read density between PrEC and LNCaP at gene promoters. In **a**–**c**, dashed lines indicate a |ΔWA| > 25, outside of which indicates a change in replication timing (red points). logFC boxplots for *later* or *earlier* genes are on the left and the right of the scatterplot, respectively. *Later* or *earlier* genes are significantly changed in expression compared to genes between |ΔWA| > 25 (asterisk, Student’s *T*-test, *p* < 2.2e−16). **d** Difference in replication timing compared to change in methylation at CpG-island promoters. Vertical dashed lines indicate a |ΔWA| > 25 (LNCaP-PrEC) and horizontal dashed lines indicate a |ΔWGBS| > 0.2 (LNCaP-PrEC). Promoters that are hypermethylated and either *later* or *earlier* are coloured in pink. **e** Comparison of expression levels (square root mean.TPM) between PrEC and LNCaP for hypermethylated and *later* gene promoters. **f** Comparison of expression levels (square root mean.TPM) between PrEC and LNCaP for hypermethylated and *earlier* gene promoters. Solid diagonal line indicates equal expression between PrEC and LNCaP; those above the line are more expressed in LNCaP and those below the line are less expressed in LNCaP. Interestingly, the hypermethylated *earlier* replicating genes that display increased expression belong to two previously described groups of CpG-island promoter hypermethylation^17^ where either, hypermethylation was associated with alternative promoter usage (*FRY, MCCC2* and *CCDC67*, red circles) or, hypermethylation of the CpG-island borders resulted in augmentation of expression in prostate cancer (*NCAM and IQGAP2*, blue circles). For boxplots, centerline indicates the median, box limits indicate upper and lower quartiles, whiskers indicate the 1.5 interquartile range and points indicate outliers

We next investigated if alterations in replication timing in cancer are associated with gene expression. Using the definition of |ΔWA| > 25, we find that more genes are replicating *later* (515) than genes that are replicating *earlier* (169) in LNCaP compared to PrEC (Fig. 3a). Genes that replicate *earlier* in LNCaP are significantly increased in expression, whereas the genes that replicate *later* are significantly repressed (Fig. 3a). We performed gene set enrichment analysis (GSEA) and observe no enrichment for *earlier* upregulated genes (Supplementary Figure 7). In contrast, genes that replicate *later* and are downregulated are enriched in terms related to cancer such as EMT, TNFα signalling, KRAS signalling, cell movement and cell proliferation (Supplementary Figure 7, Supplementary Table 1). *Later* genes in the cancer cells are also significantly enriched for tumour suppressor genes^25^ (test of equal proportions, *p* = 2.283e−06), whereas the genes replicating *earlier* are not (test of equal proportions, *p* = 0.2854). Therefore, changes in replication timing between PrEC and LNCaP cells predominantly relates to suppression of genes within cancer-related pathways in late replication.

To investigate if genes that show replication timing changes in cancer are also accompanied by chromatin remodelling, we compared the alteration in replication timing status to the enrichment of H3K27me3 and H3K4me3 at gene promoters (Fig. 3b, c). We find that genes that change to *earlier* replication timing are associated with a more active promoter environment, characterised by concordant enrichment of H3K4me3 and depletion of H3K27me3. Similarly, we find that genes that change to *later* replication timing are associated with a more repressive promoter environment, characterised by concordant depletion of H3K4me3 and enrichment of H3K27me3.

We next asked if gene promoters that are located within regions of altered replication timing in cancer also display a change in DNA methylation. Interestingly, we find distinct hypermethylation at promoter CpG-islands in cancer, regardless of the direction of replication timing change (Fig. 3d). However, a higher proportion of CpG-island promoters become hypermethylated in genes that change to a *later* replication timing (*n* = 52, 18%) relative to hypermethylated CpG-island promoters that change to an *earlier* replication timing (*n* = 12, 11%). Moreover, the genes that are hypermethylated and replicate *earlier* in LNCaP tend towards increased expression (Fig. 3e) and in contrast, genes that are hypermethylated and replicate *later* in LNCaP tend towards decreased expression (Fig. 3f). Together this data suggests that a change in replication timing is also associated with a concerted change in both gene expression and the epigenetic status of the promoter.

### Epigenetic alterations occur concordantly in the same LADs

**Fig. 4:**
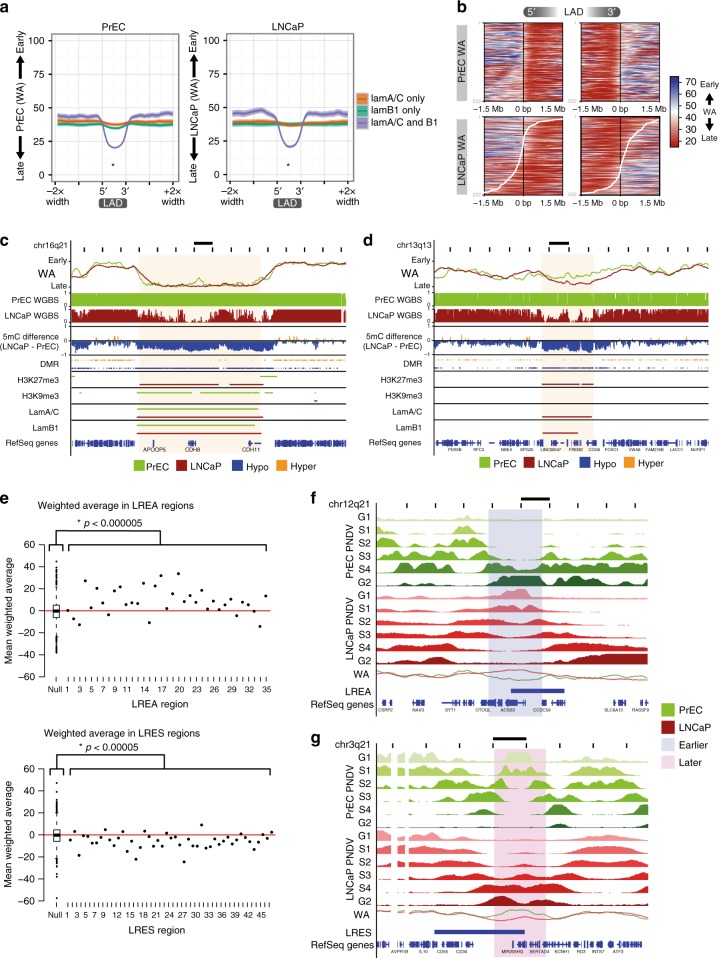
Long-range epigenetically regulated domains have altered replication timing. **a** Average plots of PrEC and LNCaP WA values over domains of lamin A/C-only, lamin B1-only or both. For both PrEC and LNCaP, LADs containing both lamin A/C and B1 are *later* than lamin A/C-only LADs (asterisk, *p* < 2.2e−16) and lamin B1-only LADs (asterisk, *p* < 2.2e−16). One-tailed Mann–Whitney–Wilcoxon tests were performed for the alternative that LADs containing both lamin A/C and B1 is ‘less’. Plots show an average line with width of shading indicating confidence intervals. **b** Heatmap of PrEC and LNCaP WA values over the boundary of PrEC LADs, ordered by degree of LAD extension (upstream of 5′ and downstream of 3′) and contraction (between 5′ and 3′) in LNCaP. The LADs used here contain both lamin A/C and lamin B1. Black lines down the centre of heatmaps represent PrEC LAD boundaries. White lines indicate the LAD boundary in LNCaP. Scale for WA is from late (red) to early (blue). Extension of the LAD boundary in LNCaP (upstream of PrEC 5′ and downstream of PrEC 3′) corresponds to these regions being significantly *later* replicating in LNCaP than PrEC (*p* < 2.2e−16, one-tailed Mann–Whitney–Wilcoxon test). Reciprocally, contraction of the LAD boundary in LNCaP (downstream of PrEC 5′ and upstream of PrEC 3′) corresponds to these regions being significantly *earlier* replicating in LNCaP than PrEC (*p* < 2.2e−16, one-tailed Mann–Whitney–Wilcoxon test). **c** Representative example of a late-replicating region in LNCaP showing maintained LADs with coordinate DNA hypomethylation, H3K27me3 gain and H3K9me3 loss (Rank 1, Supplementary Figure 8b). Scale bar represents 1 Mb. **d** Representative example of a late-replicating region in LNCaP showing coordinate LAD gain, DNA hypomethylation and H3K27me3 gain (Rank 3, Supplementary Figure 8b). Scale bar represents 1 Mb. **e** The average replication time of each LREA or LRES region is compared to a distribution of 1000 randomised LREA or LRES regions (boxplot). The significance of difference in WA distributions between regions and random is indicated (Mann–Whitney–Wilcoxon test). **f**, **g** Representative examples of overlaps between LRES and LREA, and domains of replication timing (*earlier*, *later*). Scale bar represents 1 Mb

Association with the nuclear periphery (nuclear lamina) is known to be a characteristic of late replication timing^6,9,26^, however, it is less clear how differences in nuclear lamina association between normal and cancer cells relate to alterations in cancer-associated replication timing. To address this question, we performed ChIP-seq of both lamin A/C and lamin B1 in PrEC and LNCaP. Specifically, we find that late replication timing is characterised by the presence of both lamin A/C and lamin B1 LADs rather than A/C or B1 alone (Fig. 4a), and LADs that are maintained between normal and cancer show consistent late replication timing (Supplementary Figure 8a). In contrast, LADs that are either ‘lost’ or ‘gained’ in the cancer cells show a change in their replication timing program to earlier or later replication, respectively (Supplementary Figure 8a). We were therefore interested to examine if a change in LAD boundaries between normal and cancer also relates to a change in replication timing. We plotted a heatmap of WA values over the boundaries of PrEC LADs that are shifted in LNCaP (as shown by the white curve) (Fig. 4b). We found a remarkable correlation between the borders of LADs and the transition from early replication timing outside the LAD to late replication timing within the LAD. Moreover, a shift in the LAD boundary in LNCaP, compared to PrEC, correlates with the shift where replication timing transitions from early to late, and ultimately where timing is different between the normal and cancer cell (Fig. 4b).

Since we observed differences in both DNA methylation and repressive histone modifications between LNCaP and PrEC in late replication, we next asked whether these epigenetic differences are also located at nuclear lamina and co-occur at the same loci. Previous reports have shown that DNA hypomethylation in cancer occurs in large domains that correspond to LADs or large domains of heterochromatin^27,28^. Therefore, to determine if there is a combinatorial relationship, we called broad domains of H3K27me3 and H3K9me3 and annotated the 1 kb replication timing loci for differences in the presence of histone domains, LADs and hypomethylated differentially methylated regions (DMRs) and tabulated the combinations (Supplementary Figure 8b). We find that the predominant pattern of chromatin differences at late-replicating regions in both PrEC and LNCaP is maintenance of lamina association but concomitant DNA hypomethylation, H3K27me3 enrichment, and either H3K9me3 depletion (Rank 1; 17.40%, Supplementary Figure 8b, example Fig. 4c) or H3K9me3 absence (Rank 2; 15.92%, Supplementary Figure 8c). The next most common pattern shows the acquisition of lamina association in LNCaP, a change to *later* replication and hypomethylation across the new LAD (Rank 3; 6.9%, Supplementary Figure 8b, example Fig. 4d). GSEA of the genes within these regions (Supplementary Figure 8d) indicates that Rank 1 genes are enriched in cell–cell adhesion, cell–cell junction, and adhesion molecules; Rank 2 genes in cell–cell adhesion and Rank 3 genes in neuron migration and glial cell differentiation. We find similar combinatorial histone and methylation alterations at late-replicating loci when comparing an 18-state chromHMM model (Supplementary Figure 9); the most frequent change (state 9 to 7 between PrEC and LNCaP; 15.3%) is also DNA hypomethylation, H3K9me3 loss and concordant H3K27me3 gain at late-replicating LADs (Supplementary Figure 9c, 1st row). Together, these data show that hypomethylation and heterochromatin alterations in cancer are occurring concordantly within the same late-replicating LADs.

### LRES and LREA domains have altered replication timing states

We previously identified long-range epigenetically activated LREA (*n* = 35)^17^ and silenced LRES domains (*n* = 47)^18^ in prostate cancer cells by identifying coordinated changes in gene expression and histone marks. We now ask whether LRES and LREA domains are also associated with alterations in replication timing. Compared to a random distribution of similarly sized regions, we find LREA domains are significantly distributed towards *earlier* replication and conversely, LRES domains are significantly distributed towards *later* replication in the cancer cells (Fig. 4e). Further, LRES regions more often overlap *later* domains than randomised regions (binomial test, *p* = 0.038), and similarly LREA regions more often overlap *earlier* domains (binomial test, *p* = 0.00097). Exemplary LREA regions that replicate *earlier* in LNCaP compared to PrEC, forming ectopic replication initiation zones, are shown (Fig. 4f, Supplementary Figure 10). Conversely, exemplary LRES regions that replicate *later* in LNCaP, potentially due to loss of replication initiation zones, are shown (Fig. 4g, Supplementary Figure 10). Together our data suggests that long-range domains of transcriptional alterations in cancer are also associated with concordant alterations in replication timing.

### Conservation of replication timing alterations

**Fig. 5:**
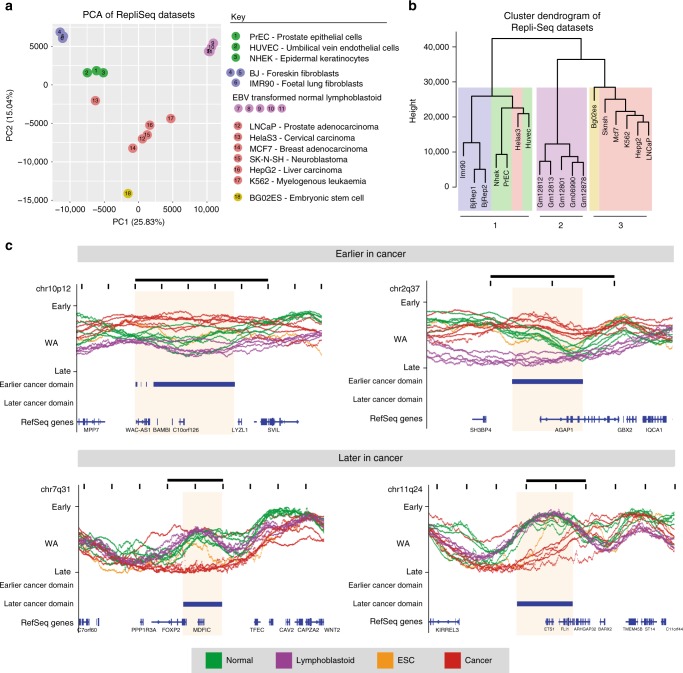
Conservation of replication timing alterations in cancer. WA values of all publically available Repli-Seq datasets, including PrEC and LNCaP, are assessed using PCA (**a**) and hierarchical clustering (Ward’s criterion) (**b**). All clusters have a clusterwise Jaccard bootstrap mean above 0.8 indicating their stability; means are 0.9399 (cluster 1), 0.8676 (cluster 2) and 0.8225 (cluster 3). Samples are identifiable by colour and number key. **c** Shown are representative examples of loci that are earlier cancer domains (ECDs) or later cancer domains (LCD) in multiple cancers compared to other cell types. Shaded boxes indicate ECDs or LCDs with logFC ≥ 1. Scale bar represents 1 Mb

We next asked whether there are associated patterns of replication timing alterations in other cancer cell types. We performed principal component analysis (PCA) and hierarchical clustering using PrEC, LNCaP and publicly available ENCODE Repli-Seq datasets^6,29^, that includes five cancer cell lines Hela S3, MCF7, SK-N-SH, HepG2 and K562, normal cultured primary cells, established fibroblast cell lines, Epstein–Barr Virus (EBV) transformed normal lymphoblastoid cells and embryonic stem cell (ESC) (Fig. 5a). In both PCA and hierarchical clustering, cell types were divided into three major clusters (Fig. 5a, b). The normal cells are clustered together, containing the normal epithelial (PrEC), epidermal and endothelial cells, as well as the fibroblasts. The normal cells are separate from the clusters containing the EBV transformed normal lymphoblasts and cancer cell lines. The closer clustering of EBV transformed normal lymphoblasts to cancer cell lines suggest the transformation process has made these cells more cancer-like. Notably, all the cancer cell lines cluster together with the exception of Hela S3. The ESC line was also found to associate with the cancer cells, potentially suggesting a progenitor-like state of cancer. Interestingly, most cancer cells assayed cluster separately to normal cells regardless of cell of origin, whereas, RNA-seq data less clearly separates normal from cancer cells (Supplementary Figure 11).

To interrogate if there are replication timing domains that are conserved across cancer types, we identified regions that displayed consistent differences in replication timing domains in the cancer datasets compared to all other non-cancer datasets. We identified 16 earlier cancer domains (ECDs) and 56 later cancer domains (LCDs). Representative examples of ECDs and LCDs across multiple cancer types are shown in Fig. 5c. Interestingly, the majority of ECDs (15/16) and LCDs (55/56) overlap regions with apparent high replication timing variation, suggesting that these loci are innately malleable, yet notably distinct in timing in cancer compared to other cell types (Supplementary Figure 12). Genes within ECDs and LCDs (Supplementary Table 2) include cancer-related genes such as *ETS1*, *FOXP2* and *BAMBI*. Notably, 46.88% of ECD and 47.37% of LCD genes have ‘lincRNA’ status based on GENCODE 19 with LCD genes being significantly enriched in lincRNAs (test of equal proportion, *p* = 0.00001128). To determine if there is potential coordinate gene function, we relaxed our domain calling cutoff (see Methods) and looked for enrichment of GSEA terms. The analyses suggest that ECD genes may play a role in cell-to-cell adhesion and LCD genes may play a role in the cell’s immune response (Supplementary Figure 13).

### Replication timing states stratify genomic rearrangements

**Fig. 6:**
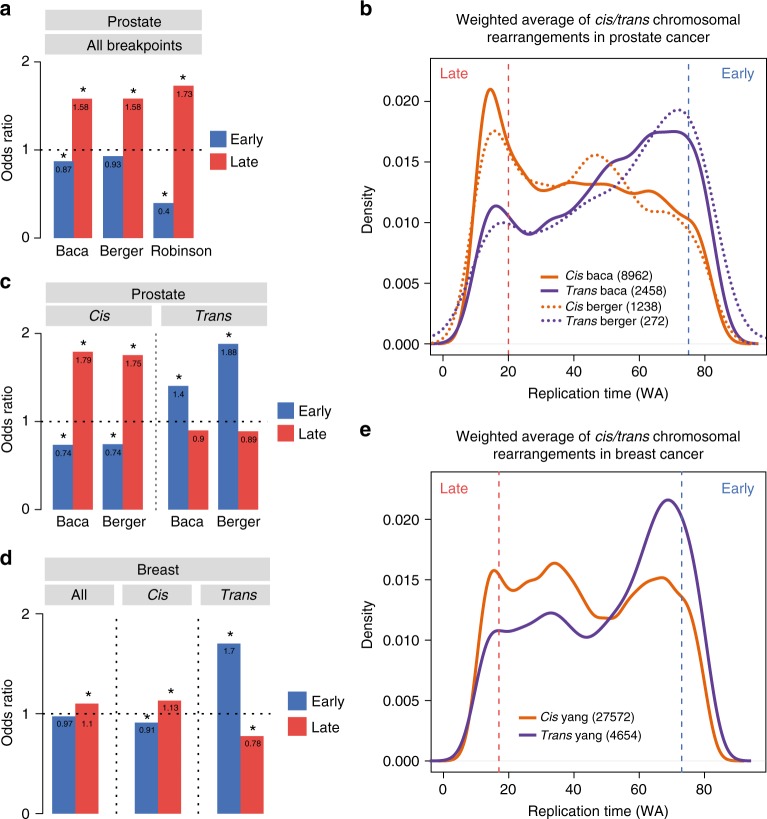
Replication timing stratifies the nature of chromosomal rearrangements. **a** Enrichment of rearrangement breakpoints for early or late replication in PrEC using public datasets^32–34^. **b** Replication timing (PrEC WA) distributions for *trans* (purple) and *cis* (orange) rearrangement breakpoints. Solid lines are distributions for Baca et al.^33^ data and dashed lines are distributions for Berger et al.^34^. Vertical red and blue dotted lines indicate late (WA < 20) or early (WA > 75) cutoffs. **c** Enrichment of rearrangement breakpoints for early or late replication separated by *cis* or *trans* status. **d** Enrichment of rearrangement breakpoints all together or separated by *cis* or *trans* status for early or late replication in MCF7 using public dataset^35^. **e** Replication timing (MCF7 WA) distributions for *trans* (purple) and *cis* (orange) rearrangement breakpoints. Vertical red and blue dotted lines indicate late (WA < 17) or early (WA > 73) cutoffs. For **a**, **c** and **d**, asterisks indicate significant enrichment (FDR < 0.05, Fisher’s exact test)

It has been previously reported that DNA hypomethylation, heterochromatin remodelling and late replication timing each separately predispose the genome to chromosomal instability in tumourigenesis^1,30,31^. Therefore, to investigate if replication timing potentially influences the nature of chromosomal rearrangements in prostate and breast tumourigenesis, we analysed publicly available clinical datasets^32–35^. We find in all prostate datasets that genomic rearrangement breakpoints are enriched in late-replicating DNA (WA < 20) and deplete in early-replicating DNA (WA > 75) in PrEC (Fig. 6a). Chromosomal rearrangements were further classified as *cis* or *trans* depending on whether they were inter- or intra-chromosomal. We find that *trans* translocations are enriched at early-replicating loci in PrEC and *cis* rearrangements at late-replicating loci in PrEC in the Baca and Berger datasets (Fig. 6b, c). We further divided the *cis* rearrangements into discrete subtypes and found inversions, deletions and long-range insertions were all enriched in late replication (Supplementary Figure 14a). To further support this finding, we used published breast cancer WGS structural variation data from Yang et al.^35^ with MCF7 replication timing data. We also observe the same trend where *trans* variants are enriched in early-replicating loci and *cis* variants are enriched in late-replicating loci (Fig. 6d, e).

We further investigated the nature of gene fusions that are commonly found in prostate cancer and asked if the genes were located in regions that displayed differences in replication timing. We examined replication timing states in PrEC and LNCaP of all the gene fusions documented in the Robinson dataset^32^. Interestingly, we found that the majority of the breakpoints occurred in regions that shared the same state of replication timing (Supplementary Figure 14b). Supplementary Dataset 1 summarises all the breakpoints in prostate cancer including those that show significant replication timing differences. Of all gene fusions assayed, *TMPRSS2-ERG* located on chromosome 21q22.2 was the most common (209/4122) fusion found in patients. Strikingly, in 195/209 fusion events, the 3′ *ERG* translocation points were located in a domain that changes replication timing from early to late S-phase in LNCaP (Supplementary Figure 14b). Supplementary Figure 14c shows the domain of replication timing change that harbors the *ERG* locus. Taken together our data shows that the replication timing status of a locus is associated with increased susceptibility to genomic rearrangement and notably, bias loci towards either *cis* or *trans* translocations.

## Discussion

The major focus of this study was to address whether there are replication timing differences in cancer and if so, to investigate what was the association between replication timing and alterations to the cancer epigenome and genome. Here, we make the noteworthy discovery that replication timing and the opposing remodelling of the epigenome, in early vs. late replication, is associated with the mode of chromosomal rearrangements in cancer. We demonstrate that genomic regions that undergo long-range epigenetic deregulation in cancer also show concordant differences in replication timing. Notably, late-replicating regions in prostate and breast cancer cells display a remarkable reduction of DNA methylation, and a switch in heterochromatin features from H3K9me3-marked constitutive to H3K27me3-marked facultative heterochromatin. Our data support a model where alterations in epigenetic remodelling in cancer cells in early and late replicating loci provide increased probability for *cis* or *trans* chromosomal rearrangements based on the nuclear spatial context.

The replication timing program is known to be re-organised during cellular differentiation and reflects cellular identity^11,14,15,21^. Therefore, it was interesting to find that the replication timing profiles between different cancer cell types are more similar to each other than to non-cancer cells, suggesting that there may be shared domains that commonly display alterations in replication timing in tumourigenesis. These shared domains may have potential functional relevance to cancer, as we find enrichment of genes involved in cancer-related pathways, such as cell-to-cell adhesion and immunological signatures, and also enrichment in genes with lincRNA status raising the possibility of important regulatory functions that are potentially altered in cancer^36^.

It has previously been proposed that replication timing represents a higher-order functional unit^9,29^, however our data now suggests that timing is also a unit for epigenomic deregulation during tumourigenesis. We find transcriptional and epigenetic alterations within altered replication timing regions between normal and cancer cells. We further find that domains of replication timing alterations in prostate cancer cells correlate with previously observed domains of long-range transcriptional and epigenetic remodelling for both LRES^18^ and LREA^17^ regions, whereby *earlier* domains become more active and *later* domains become more repressive. This directional relationship between replication timing and gene expression has been observed in development and differentiation^11,14,15^. For example in human cell lines, Rivera-Mulia et al.^11^ report that developmental shifts to *later* timing precedes transcriptional down-regulation and developmental shifts to *earlier* timing follows transcriptional up-regulation for the majority of replication timing switching genes, suggesting that replication timing may be both a driver and a passenger of transcription. In our study, we find that *later* replicating genes are primarily down-regulated, and show cancer-related gene ontology. We therefore speculate that a change to *later* timing in tumourigenesis potentially precedes down-regulation of these cancer-associated genes. Whilst previous studies^37,38^ have shown that replication timing can impose particular chromatin states, more work is required to establish whether epigenetic changes come first and are prior too or occur as a result of a change in replication timing in cancer.

There has been no integrative study between replication timing and epigenomic changes in normal and cancer cells in the same cell system. Pairwise associations between replication timing and epigenetic features have been observed during mouse ES cell differentiation or between mostly disjoint normal human cell lines from different tissue origins^12,13,26–28,39^. Here, using an integrative approach we now identify that the most widespread combinatorial epigenomic alteration between normal and prostate cancer cells is DNA hypomethylation, accompanied with a switch from H3K9me3-constitutive to H3K27me3-facultative heterochromatin within lamina-bound late-replicating regions. H3K27me3 and DNA methylation are generally antagonistic and commonly switch in cancer cells^28,40^ and DNA hypomethylation in mouse embryonic fibroblasts leads to redistribution of H3K27me3^41^, suggesting a direct relationship between loss of DNA methylation and gain of H3K27me3. H3K9me3 loss may also directly contribute to DNA hypomethylation, as H3K9me3 is required to enhance the activity of UHRF1 and consequently DNMT1^42,43^. Our findings suggest that there is a highly co-ordinated alteration of the cancer epigenome that occurs exquisitely in genomic regions that replicate late in S-phase.

Finally, we observe an association between chromosomal rearrangements and replication timing, with late-replicating regions prone to *cis* rearrangements and early-regions to *trans* rearrangements in both breast and prostate cancer. Previous studies^1,44–46^ have reported associations of chromosomal rearrangements with both early and late replication, but not in relation to the altered state of the epigenome. Of note, Morganella et al.^46^ identified all breast cancer rearrangements to be associated with early replication timing, whereas, De et al.^1^ identified that large deletions are enriched for late replication while large amplifications are enriched for early replication. These reports are in contrast to our study where we find a clear bias towards late replication for most rearrangements other than translocations. The differences could be due to differential DNA repair pathway usage between early and late regions of the nucleus^47,48^ and the repair pathways active within the different cancer types.

**Fig. 7:**
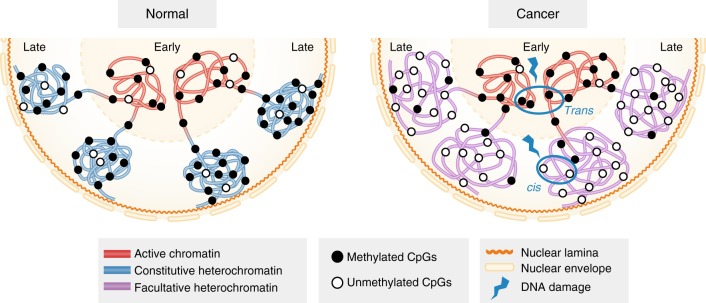
Late replication timing is more sensitive to genetic and epigenetic damage. Replication timing is spatially organised within the nucleus. Transcriptionally active early-replicating loci are often close in proximity with each other in transcriptional hubs towards the nuclear centre^51,78,79^. Transcriptionally inactive late-replicating loci are typically heterochromatin, condensed and localised to the nuclear periphery and nuclear lamina^51,78,79^. In prostate and breast cancer cells, early-replicating loci remain transcriptionally active, however, late-replicating loci switch from a methylated H3K9me3-marked constitutive heterochromatin state to a hypomethylated H3K27me3-marked facultative heterochromatin state, and potentially more susceptible to DNA damage and/or error-prone repair leading to an increase in chromosomal rearrangements. We speculate that if a DNA break occurs in late replication it is more likely to be repaired in *cis* as late-replicating loci are more self-contained and located towards the nuclear periphery. In contrast, if a break occurs in early replication, this is more likely to result in *trans* translocations, as there is increased potential for interchromosomal interactions within structures like transcriptional hubs in the nuclear centre

Our combinatorial epigenome and replication timing data leads us to propose a model to explain how replication timing status and associated epigenetic alterations may influence the nature of chromosomal cancer rearrangements (Fig. 7). Hypomethylation and loss of H3K9 methyltransferases have been separately linked to increased genomic instability in cancer^30,31^. However, our data shows that late-replicating loci in cancer are both hypomethylated and switched from a H3K9me3-marked constitutive heterochromatin state to a H3K27me3-marked facultative heterochromatin state. We suggest that it is the combination of epigenetic remodelling in the context of the replication timing state that is associated with increased chromosomal rearrangements. In particular, we hypothesise that the switch to facultative heterochromatin may sensitise late-replicating regions to DNA damage and/or error-prone repair. Previous 3D studies suggest that the chance of a translocation occurring is proportional to the degree of chromatin interaction between two loci and interactions occur more frequently between loci within the same chromosome territory or between adjacent territories than between distant territories^49–51^. Interphase chromosomes are organised such that early-replicating loci are located towards the nuclear centre and late-replicating loci are located towards the nuclear periphery (lamina-bound) (Fig. 7)^9,21,26^. We therefore propose that bias towards *cis* or *trans* chromosomal rearrangement is related to the spatial and temporal positioning of early-replicating compared to late-replicating loci. As late-replicating loci are more self-contained^52^, we suggest that DNA breaks are more likely to involve *cis* rearrangements. In contrast as early-replicating loci are more interactive^52,53^, DNA breaks occurring in regions replicating in early S-phase are more likely to result in *trans* translocations.

Our model is also supported by studies that show copy number aberration breakpoints generally have the same replication timing and interact long-range^1^, and that translocation partners are required to be within the same spatial area, transcription^54^ or replication factory^55^, before translocation can occur. A pertinent example for  prostate cancer is the *TMPRSS2-ERG* gene fusion that occurs in ~50–80% of all prostate cancer^56^. Studies show that nuclear spatial proximity between *TMPRSS2* and *ERG* is a determining factor of fusion frequency^57^. Furthermore, spatial proximity can be induced through activation of the genes by the androgen receptor (AR) under testosterone (DHT) treatment, which works to increase spatial proximity by targeting *TMPRSS2* and *ERG* to the same replication factory^55,57^. In conclusion, our combinatorial data analysis supports a paradigm where epigenetic deregulation between early and late replication can modulate the mutational landscape and underpin long-range epigenetic deregulation of the cancer genome.

## Methods

### Cell culture

All cancer cell lines were purchased from ATCC (USA) and cultured in our lab following standard protocols. LNCaP human prostate cancer cells (ATCC #CRL-1740) were maintained in T-medium (Gibco, Formula no. 02-0056DJ) supplemented with heat-inactivated foetal bovine serum (10%, Gibco, 16000-044), L-Glutamine (2 mM) (Gibco, #25030-081) and Penicillin/Streptomycin (50 units/50 μg) (Gibco, #15070-063). MCF7 human breast cancer cells (ATCC #HTB-22) were maintained in RPMI-1640-based medium containing 5% (v/v) foetal bovine serum. PrEC normal human prostate epithelial cells (Cambrex Bio Science, #CC-2555, tissue acquisition number 13683) were maintained in prostate epithelial cell growth medium (PrEGM, Lonza, #CC-3165) supplemented with SingleQuots growth supplements (Lonza, #CC-4177).

### Repli-Seq data generation and processing

BrdU-labelled DNA was generated as previously described^6,20^. Briefly, cells were labelled with BrdU (50 μM, Sigma, #B5002) for 2 h. Labelled cells were sorted into 6 fractions across the cell cycle (G1b, S1, S2, S3, S4, G2M) as per protocol on the FACS AriaII. DNA extraction and BrdU-labelled DNA immunoprecipitation were performed with anti-BrdU antibody (40 μL of 25 μg mL^−1^, BD Pharmingen, #555627). Validation of BrdU immunoprecipitation was carried out using qPCR on known Early (*BMP1*) and Late (*DPPA2*) loci (Supplementary Figure 1a, b, Supplementary Table 3). ssDNA was reconstituted for the complementary DNA strand using Klenow extension with random hexamers (Random Primers DNA Labeling System, Invitrogen #18187-013) using 10 ng of ssDNA input and a 2 h incubation. Reconstituted dsDNA was rechecked for enrichment of known early and late loci using qPCR (Supplementary Figure 1c). Klenow-treated products were sonicated using a Sonifier 250 probe sonicator. 15 μL of Klenow-treated dsDNA was sent to University of Southern California Epigenome Centre Data Production Facility for 50 bp single-end sequencing on the Illumina HiSeq 2000. The DNA amounts and full sequencing details can be found in Supplementary Table 4.

Replication timing WA scores were calculated according to Hansen et al.^6^ with slight modifications. Repli-Seq fractions were mapped to hg19 using bowtie^58^ (v1.1.0). To avoid bias from duplications or repeats, read densities were calculated in 150 bp intervals and intervals were excluded from further analysis if they contained greater than 20 reads per 150 bp window. Read densities were calculated in 50 kb sliding windows at 1 kb intervals across the remaining genomic regions, excluding chrY and chrM. Read densities were normalised to reads/counts per million and 50 kb windows with low coverage (5 reads/counts per million) were removed. To account for variation in sequencing coverage, mapability and cell-type specific copy-number variations, the remaining 50 kb window reads/counts per million values in all 6 fractions of a given sample were converted to a percentage of total signal at each 1 kb locus called percent-normalised density values (PNDV) (Supplementary Figure 1d). The PNDV value represents the percentage of replication occurring within a particular timing fraction at a given 1 kb locus. PNDV values were then converted into a single replication timing WA score per 1 kb loci using the following formula: weighted average = (0.917*G1) + (0.750*S1) + (0.583*S2) + (0.417*S3) + (0.250*S4) + (0*G2). The formula for this transformation was obtained from the ENCODE method for ‘Replication Timing by Repli-Seq’. WA values represent the time of replication, where a higher WA was indicative of an earlier time of replication.

Repli-Seq was performed in duplicate for each cell line. Each sample was processed independently up to and including calculation of the WA value. WA values for replicates of LNCaP and PrEC are highly correlated (*r*^2^ values > 0.99) (Supplementary Figure 1e). Duplicate WA values per cell line were then averaged and used for downstream analysis. The distributions of WA scores were comparable to the WA distributions in other normal and cancer cell Repli-Seq datasets (Supplementary Figure 1f). Early- and late-replicating regions were defined as those regions in the top and bottom 10% of WA scores in both cell lines. This definition gives upper and lower limits of 75 and 20 respectively for PrEC and LNCaP (i.e. early regions have WA > 75 and late regions WA < 20). WA thresholds for a change in timing were defined by the following process: differences in WA between replicates of the same cell line are representative of random noise and therefore can be used as an empirical null distribution for the hypothesis test that the WA difference between LNCaP and PrEC is equal to zero. The maximum observed difference, in our data, between replicates is |ΔWA| = 23 (Supplementary Figure 1h). To be conservative, we chose a cut-off of |ΔWA| > 25 as a value that occurs infrequently (or never) by chance under the null. Differences in WA that are larger than ±25 ΔWA are therefore considered to show a robust change in replication timing. To identify domains of loci with changed replication timing, we merged all loci within 50 kb that had |ΔWA| > 25. On average, altered replication timing domains were ~295 kb wide. For MCF7, the top 10% of early- and late-replicating loci gives upper and lower WA limits of 73 and 17.

### Whole genome bisulphite sequencing and processing

PrEC and LNCaP WGBS libraries were prepared using the Illumina Paired-end DNA Sample Prep Kit (discontinued, Illumina, CA, USA). Briefly, genomic DNA was extracted from cells using QIAamp Mini Kit (QIAGEN, #51304). 1 μg of input DNA was spiked with unmethylated lambda DNA (0.5%) (Promega, #D1521). DNA was sonicated to produce DNA fragments of around 250 bp in length, end-repaired using the Paired-End Sample Prep kit (Illumina #PE102-1001) and cleaned using QIAquick PCR Purification kit (QIAGEN, #28104). A-tailing and adapter ligation were performed according to the Illumina protocol. The adapter-ligated DNA was gel size selected (260–330 bp) using Qiagen Gel extraction kit (Qiagen, #28704). Bisulphite treatment was carried out as previously described^59^, with the bisulphite reaction performed for 4 h at 55 °C. Converted libraries were enriched in five independent PCR reactions for 10 cycles using PfuTurbo Cx Hotstart DNA polymerase (Stratagene, #STG600410). The five independent reactions were pooled and purified using the MinElute PCR Purification Kit (QIAGEN, #28004). Paired-end 100 bp sequencing was performed for each library on the Illumina HiSeq 2500 platform. The MCF7 WGBS library was performed using the CEGX TrueMethyl Whole-Genome kit (v2.1).

WGBS libraries were processed as previously described^60^ (see Supplementary Methods). DMRs were called from PrEC and LNCaP WGBS data using the package MethPipe^61^ (v3.4.2). PMDs were called from LNCaP WGBS data using MethPipe.

### RNA-seq data generation and processing

For PrEC and LNCaP, total RNA in biological triplicates was spiked with external controls (ERCC RNA spike-in Mix, Thermo Fischer, #4456740) and libraries were constructed with the Illumina TruSeq Stranded mRNA sample preparation kit. Genes with fold change ±1.5 and FDR < 0.01 were considered as significantly altered. RNA-seq datasets were processed as previously described^16^ (see Supplementary Methods). RNA-seq processed for PCA and hierarchical clustering was performed with a modified version of the in-house RNA-seq pipeline (see Supplementary Methods). Datasets were normalised using ERCC controls before calculating logCPMs (edgeR^62^).

### ChIP-seq assay and processing

ChIP assays were performed as previously described^17,18,63,64^ for the following histone marks: H3K4me3 (Abcam, #ab8580), H3K4me1 (Active Motif, #39297), H3K36me3 (Abcam, #ab9050), H3K27ac (Active Motif, #39133), H2AZac (Abcam, #ab18262), H3K9ac (Millipore, #06-599) and H3K27me3 (Millipore, #07-449). ChIP of H3K9me3 (Diagenode, #C15500003) was performed as previously described^65^. We performed lamin ChIP assays in PrEC and LNCaP as previously described^66^ for both Lamin B1 (Abcam, #ab16048) and Lamin A/C (Santa Cruz, #sc7292). Each ChIP assay was validated by qPCR against an IgG control and enrichment above input. Libraries were prepared with the Illumina TruSeq Chip Library Prep Kit and sequenced on an Illumina HiSeq 2500.

Sequencing data was processed as previously described^17,63^. Briefly, ChIP-seq reads were aligned to hg19 using bowtie^58^ (v1.1.0) allowing up to 3 mismatches, discarding ambiguous and clonal reads. All histone ChIP-seq peaks were called using PeakRanger^67^ (v1.16). Broad domains of lamins (LADs), H3K9me3 and H3K27me3 were called using the enriched domain detector (EDD) for identification of wide genomic enrichment domains^68^.

### DNase1 hypersensitivity assay

Cells (7 × 10^6^ per sample) were scraped, centrifuged and washed with PBS. Cell pellets were resuspended in nuclear extraction buffer (10 mM Tris–HCl pH7.4, 12.5 mM NaCl, 3 mM MgCl, 0.1 mM EDTA, 0.5% IGEPAL) and dounced until nuclei were visible under light microscope with 0.4% Trypan Blue staining. DNase1 (Roche, #04716728001) was added to nuclei pellets of LNCaP (24 U) and PrEC (12 U) and incubated at 37 °C for 15 min. DNase1 reactions were terminated by the addition of 36 mM EDTA and Proteinase K was added before incubating at 55 °C overnight. DNA was purified by phenol-chloroform extraction and ethanol precipitation. Samples were separated using electrophoresis on a 1% agarose gel. 100–300 bp sections were excised and purified using the QIAquick Gel Extraction kit. Libraries were prepared with the Illumina TruSeq Chip Library Prep Kit and sequenced on an Illumina HiSeq 2000.

### Quantification of epigenetic marks over replication timing loci

We defined chromatin mark occupancy and methylation averages for the 1 kb wide blocks produced in the Repli-Seq data processing. Chromatin-mark-occupied 1 kb blocks were defined as any 1 kb block that overlapped a called ChIP-seq peak. *Gain* of chromatin mark is defined as the same 1 kb block overlapping a ChIP peak in LNCaP and not in PrEC, and the reciprocal for *loss*. Methylation values were averaged over the same 1 kb blocks using the overlapMeans function within R package aaRon (https://github.com/astatham/aaRon.git). Hypomethylation was defined as a loss of >0.2 between PrEC and LNCaP, and hypermethylation was defined as a gain of >0.2 between PrEC and LNCaP. For hypomethylation, we only considered loci with methylation values of at least 0.2 in PrEC, and for hypermethylation, we only considered loci with methylation values below 0.8 in PrEC.

### Quantification of epigenetic marks over promoters

Promoters were defined as ±1000 bp around the transcription start site from the GENCODE 19 reference transcriptome. CpG-island promoters were defined as any promoter (2 kb) that overlapped with a CpG-island. Early- and late-replicating genes were defined by calculating the average WA score over the promoter. The genes with average WA scores >75 or <20 were labelled as early and late-replicating, respectively. Promoter-centric ChIP-seq enrichment for H3K4me3 and H3K27me3 was calculated by counting the number of reads within the promoter region using the Repitools R package. Fold changes (logFC) were computed as the log2 ratio of normalised counts per promoter using the edgeR^62^. Methylation values were averaged over promoter regions using the overlapMeans function within R package aaRon (https://github.com/astatham/aaRon.git).

### Genomic annotation

The CpG-islands are from Gardiner-Garden and Frommer (1987), downloaded from UCSC^69^. We annotated the CpG-islands to promoters based on overlaps to promoter regions from GENCODE 19 genes. For hypomethylation, we only considered CpG-islands with methylation values of at least 0.2 in PrEC, and for hypermethylation, we only considered CpG-islands with methylation values below 0.8 in PrEC. CpG-island shores are defined as the regions within 2 kb either side of a CpG-island. Exons and introns were called per transcript using the GenomicFeatures^70^ package in R, and merged if overlapping. Intron regions were retained if they did not intersect an exonic region. 5′ and 3′ UTRs were also called using the GenomicFeatures package, and merged if overlapping. Intergenic regions were defined as the gaps between the other elements.

### Statistical tests

For genomic interval overlaps and genomic rearrangement overlaps, we modified the LOLA^71^ package to perform a two-sided Fisher's exact test and reports significance using ‘BH’ FDR value. Differences between percentages of epigenetic elements in Early or Late timing were assessed using the two-sample test of equal or given proportions (prop.test in R). The Student’s *T*-test was used to test for significant difference between two groups of logFC values as produced from edgeR processing. The Mann–Whitney–Wilcoxon test was used for 2-group non-parametric comparisons, and the one-tailed test was used where a directional difference between the groups was expected. Unless otherwise stated, statistical tests were two-sided.

Creating a randomised set of LRES and LREA domains for testing statistical association of domains to early or late replication: Random genomic regions were generated in a three-stage process: first, a chromosome was selected at random, second, the start point of the region was randomly generated from a uniform distribution between 1 and the length of the chromosome, and last, the length of the region to be generated by sampling at random from the known lengths of LREA or LRES regions. If the random region generated did not fit on the chromosome, it was discarded and the process repeated. In this way, we generated 1000 regions across the genome that were distributed in length similarly to the LREA and LRES regions. We computed the WA for each random region and compared this empirical null distribution to the distribution of WA values for LREA and LRES using the Mann–Whitney–Wilcoxon test. We used an exact binomial test to examine the significance of overlap between LRES or LREA regions and *earlier* or *later* domains. We used the proportion of randomised regions overlapping *earlier* or *later* domains as the hypothesised probability of success in the binomial test.

### Profile plots

We used genomation^72^ to calculate average WA scores over regions of interest, which were divided into 50 bins per region. We then used ggplot^73^ to plot the average WA scores across all regions for each bin with standard error and confidence intervals.

### Lamina boundary heatmaps

We calculated the bp distance from the PrEC LAD 5′ or 3′ boundary to the nearest LNCaP LAD 5′ or 3′ boundary, respectively. Negative and positive distances denote that the LNCaP boundary is respectively upstream or downstream of the PrEC boundary. Heatmaps of WA values are centred on PrEC LAD boundaries, and are ordered by decreasing distance to the nearest LNCaP boundary. This was performed for LADs containing both lamin A/C and lamin B1, and LADs that overlapped between PrEC and LNCaP.

### ChromHMM of heterochromatin marks and replication domains

ChromHMM^74^ was used to create a 18-state model from 7 marks for PrEC and LNCaP. The inputs were: EDD called bed files for H3K27me3, H3K9me3, lamin A/C and lamin B1; unmethylated or ‘hypo’-methylated (HMR) bed files called by MethPipe^61^; and early and late 1 kb bed files called using the cut-offs WA > 75 and WA < 20, respectively. Binary files were created using BinarizeBed with the ‘-peaks’ option. 8–30 models were created with LearnModel using default parameters. We chose the 18-state model as it displayed the most informative states while maintaining a manageable number of pairwise state transitions for interpretability. Pairwise combinations were counted per 200 bp bin (default bin size for ChromHMM) along the genome.

### Conserved replication timing alterations in cancer

WA values from all publically available Repli-Seq datasets and our datasets were quantile-normalised prior to performing PCA and hierarchical clustering. PCA was performed on 1 kb loci that were present in all datasets using the R function prcomp with default parameters. Hierarchical clustering was performed using the hclust function in R with the Ward’s criterion (‘ward.D2’) method. Distance matrix for clustering was computed using the dist function in R and the Euclidean method. Cluster groups were defined using rect.hclust from the stats package in R. Cluster bootstrapping was performed using clusterboot from the ‘fpc’ package in R. The same PCA and clustering was performed on PrEC, LNCaP and public RNA-seq datasets using quantile-normalised and replicate averaged logCPM values. To find conserved regions of changed timing in cancer (LNCaP, MCF7, SK-N-SH, HepG2, K562, Hela S3) compared to all other Repli-Seq datasets (see Fig. 5a), WA values were quantile-normalised and scaled before using limma^75^ to find regions of difference. We filtered for regions that were significant (FDR-corrected *p* < 0.05) with a logFC ≥ 1. These regions were merged if they were within 50 kb of each other to give the final set of ECDs and LCDs. We further filtered ECDs and LCDs through overlap with high and low replication timing variation regions. These high/low variation regions were defined as the top and bottom 20% of 1 kb loci based on scores outputted by the ‘var’ function in R. Repli-Seq scores per loci from all was used as input for ‘var’ to calculate a score per loci. We further separated ECDs and LCDs into ones that associate with replication timing of ESC. We classified a region as associated with ESC if the difference between the averaged replication timing score of cancer to ESC was less than 0.5 for ECDs or more than −0.5 for LCDs (quantile-normalised and scaled WA scores).

### Gene set enrichment analysis for genes in ECDs and LCDs

We were unable to call significant terms from GSEA with the stringent cutoffs initially used to define ECD and LCDs (logFC ≥ 1, see above) due to the restricted number of genes obtained. We relaxed our domain calling cutoff to logFC > 0 to obtain a less stringently defined but larger list of genes for GSEA analysis. We used a hyper-geometric test to scan the MolSigDB v6.0^76,77^ for gene sets with statistically significant overlap with genes found within ECDs and LCDs. More specifically, we computed the overlap between the MolSigDB gene sets and our set of genes and compared what would be expected by chance if equivalent number of genes were drawn uniformly at random from the background set of genes. We report statistically significant enrichments with Bonferroni-corrected *p* < 0.05.

### External data

For prostate cancer breakpoints, we used the Baca et al.^33^, Berger et al.^34^ and Robinson et al.^32^ datasets. Publicly available Repli-Seq datasets used in this study were downloaded from ENCODE data portal (https://www.encodeproject.org/matrix/?type=Experiment&assay_title=Repli-seq). These datasets were created by the University of Washington ENCODE group^14,29^. Raw sequence data were downloaded from UCSC, mapped to hg19 and processed in the same manner as our own Repli-Seq data as described above. Publicly available RNA-seq datasets used in this study were downloaded from ENCODE (Supplementary Table 5). HMEC and MCF7 ChIP-seq data was downloaded from ENCODE (Supplementary Table 5). HMEC WGBS was downloaded from GSE29127.

### Reporting summary

Further information on experimental design is available in the Nature Research Reporting Summary linked to this article.

### Code availability

All software used is published and/or in the public domain. Custom R code is available at https://github.com/clark-lab/Replication-Timing.

## Supplementary information

Supplementary Information

Description of Additional Supplementary Files

Supplementary Data 1

Reporting Summary

## Data Availability

The data that support the findings of this study are available from the corresponding author upon request: raw and processed. Repli-Seq and ChIP-seq data are available from NCBI Gene Expression Omnibus (GEO) under accession number GSE98732. RNA-seq data GEO accession number GSE73784. ChIP-seq data GEO accession numbers GSE38685, GSE57498, GSE73785 and GSE76337. PrEC and LNCaP WGBS data GEO accession number. Prostate cancer WGBS GEO accession number GSE104789. A summary of the new and existing data used in this manuscript for PrEC and LNCaP can be found in Supplementary Table 6. A reporting summary for this article is available as a Supplementary Information file.

## References

[1] De S, Michor F (2011). DNA replication timing and long-range DNA interactions predict mutational landscapes of cancer genomes. Nat. Biotechnol..

[2] Hodgkinson A, Chen Y, Eyre-Walker A (2012). The large-scale distribution of somatic mutations in cancer genomes. Hum. Mutat..

[3] Woo YH, Li WH (2012). DNA replication timing and selection shape the landscape of nucleotide variation in cancer genomes. Nat. Commun..

[4] Schuster-Bockler B, Lehner B (2012). Chromatin organization is a major influence on regional mutation rates in human cancer cells. Nature.

[5] Alabert C, Groth A (2012). Chromatin replication and epigenome maintenance. Nat. Rev. Mol. Cell Biol..

[6] Hansen RS (2010). Sequencing newly replicated DNA reveals widespread plasticity in human replication timing. Proc. Natl. Acad. Sci. U.S.A..

[7] Rhind N, Gilbert DM (2013). DNA replication timing. Cold Spring Harb. Perspect. Med..

[8] Julienne H, Zoufir A, Audit B, Arneodo A (2013). Human genome replication proceeds through four chromatin states. PLoS Comput. Biol..

[9] Pope BD (2014). Topologically associating domains are stable units of replication-timing regulation. Nature.

[10] Dileep V (2015). Topologically associating domains and their long-range contacts are established during early G1 coincident with the establishment of the replication-timing program. Genome Res..

[11] Rivera-Mulia JC (2015). Dynamic changes in replication timing and gene expression during lineage specification of human pluripotent stem cells. Genome Res..

[12] Aran D, Toperoff G, Rosenberg M, Hellman A (2011). Replication timing-related and gene body-specific methylation of active human genes. Hum. Mol. Genet..

[13] Suzuki M (2011). Late-replicating heterochromatin is characterized by decreased cytosine methylation in the human genome. Genome Res..

[14] Hiratani I (2010). Genome-wide dynamics of replication timing revealed by in vitro models of mouse embryogenesis. Genome Res..

[15] Hiratani I (2008). Global reorganization of replication domains during embryonic stem cell differentiation. PLoS Biol..

[16] Taberlay PC (2016). Three-dimensional disorganization of the cancer genome occurs coincident with long-range genetic and epigenetic alterations. Genome Res..

[17] Bert SA (2013). Regional activation of the cancer genome by long-range epigenetic remodeling. Cancer Cell.

[18] Coolen MW (2010). Consolidation of the cancer genome into domains of repressive chromatin by long-range epigenetic silencing (LRES) reduces transcriptional plasticity. Nat. Cell Biol..

[19] Ryba, T. et al. Abnormal developmental control of replication-timing domains in pediatric acute lymphoblastic leukemia. *Genome Res.* **22**, 1833–1844 (2012). 10.1101/gr.138511.112 PMC3460179 22628462

[20] Ryba T, Battaglia D, Pope BD, Hiratani I, Gilbert DM (2011). Genome-scale analysis of replication timing: from bench to bioinformatics. Nat. Protoc..

[21] Ryba T (2010). Evolutionarily conserved replication timing profiles predict long-range chromatin interactions and distinguish closely related cell types. Genome Res..

[22] Bender S (2013). Reduced H3K27me3 and DNA hypomethylation are major drivers of gene expression in K27M mutant pediatric high-grade gliomas. Cancer Cell.

[23] McDonald OG (2017). Epigenomic reprogramming during pancreatic cancer progression links anabolic glucose metabolism to distant metastasis. Nat. Genet..

[24] Zhou W (2018). DNA methylation loss in late-replicating domains is linked to mitotic cell division. Nat. Genet..

[25] Zhao M, Sun J, Zhao Z (2012). TSGene: a web resource for tumor suppressor genes. Nucleic Acids Res..

[26] Peric-Hupkes D (2010). Molecular maps of the reorganization of genome–nuclear lamina interactions during differentiation. Mol. Cell.

[27] Berman BP (2012). Regions of focal DNA hypermethylation and long-range hypomethylation in colorectal cancer coincide with nuclear lamina-associated domains. Nat. Genet..

[28] Hon GC (2012). Global DNA hypomethylation coupled to repressive chromatin domain formation and gene silencing in breast cancer. Genome Res..

[29] Thurman RE, Day N, Noble WS, Stamatoyannopoulos JA (2007). Identification of higher-order functional domains in the human ENCODE regions. Genome Res..

[30] Chen TP (2007). Complete inactivation of DNMT1 leads to mitotic catastrophe in human cancer cells. Nat. Genet..

[31] Peters AH (2001). Loss of the Suv39h histone methyltransferases impairs mammalian heterochromatin and genome stability. Cell.

[32] Robinson D (2015). Integrative clinical genomics of advanced prostate cancer. Cell.

[33] Baca SC (2013). Punctuated evolution of prostate cancer genomes. Cell.

[34] Berger MF (2011). The genomic complexity of primary human prostate cancer. Nature.

[35] Yang L (2013). Diverse mechanisms of somatic structural variations in human cancer genomes. Cell.

[36] Ransohoff JD, Wei Y, Khavari PA (2018). The functions and unique features of long intergenic non-coding RNA. Nat. Rev. Mol. Cell Biol..

[37] Lande-Diner L, Zhang J, Cedar H (2009). Shifts in replication timing actively affect histone acetylation during nucleosome reassembly. Mol. Cell.

[38] Zhang J, Xu F, Hashimshony T, Keshet I, Cedar H (2002). Establishment of transcriptional competence in early and late S phase. Nature.

[39] Sadaie M (2013). Redistribution of the Lamin B1 genomic binding profile affects rearrangement of heterochromatic domains and SAHF formation during senescence. Genes Dev..

[40] Reddington JP, Sproul D, Meehan RR (2014). DNA methylation reprogramming in cancer: does it act by re-configuring the binding landscape of Polycomb repressive complexes?. Bioessays.

[41] Reddington JP (2013). Redistribution of H3K27me3 upon DNA hypomethylation results in de-repression of Polycomb target genes. Genome Biol..

[42] Rothbart SB (2012). Association of UHRF1 with methylated H3K9 directs the maintenance of DNA methylation. Nat. Struct. Mol. Biol..

[43] Liu X (2013). UHRF1 targets DNMT1 for DNA methylation through cooperative binding of hemi-methylated DNA and methylated H3K9. Nat. Commun..

[44] Shugay M, Ortiz de Mendibil I, Vizmanos JL, Novo FJ (2012). Genomic hallmarks of genes involved in chromosomal translocations in hematological cancer. PLoS Comput. Biol..

[45] Koren A (2012). Differential relationship of DNA replication timing to different forms of human mutation and variation. Am. J. Hum. Genet..

[46] Morganella S (2016). The topography of mutational processes in breast cancer genomes. Nat. Commun..

[47] Chakraborty A (2016). Classical non-homologous end-joining pathway utilizes nascent RNA for error-free double-strand break repair of transcribed genes. Nat. Commun..

[48] Lemaitre C (2014). Nuclear position dictates DNA repair pathway choice. Genes Dev..

[49] Meaburn KJ, Misteli T, Soutoglou E (2007). Spatial genome organization in the formation of chromosomal translocations. Semin. Cancer Biol..

[50] Branco MR, Pombo A (2006). Intermingling of chromosome territories in interphase suggests role in translocations and transcription-dependent associations. PLoS Biol..

[51] Yaffe E, Tanay A (2011). Probabilistic modeling of Hi-C contact maps eliminates systematic biases to characterize global chromosomal architecture. Nat. Genet..

[52] Lieberman-Aiden E (2009). Comprehensive mapping of long-range interactions reveals folding principles of the human genome. Science.

[53] Brown JM (2008). Association between active genes occurs at nuclear speckles and is modulated by chromatin environment. J. Cell Biol..

[54] Ugarte GD (2015). Wnt signaling induces transcription, spatial proximity, and translocation of fusion gene partners in human hematopoietic cells. Blood.

[55] Coll-Bastus N, Mao X, Young BD, Sheer D, Lu YJ (2015). DNA replication-dependent induction of gene proximity by androgen. Hum. Mol. Genet..

[56] Tomlins SA (2005). Recurrent fusion of TMPRSS2 and ETS transcription factor genes in prostate cancer. Science.

[57] Mani RS (2009). Induced chromosomal proximity and gene fusions in prostate cancer. Science.

[58] Langmead B, Trapnell C, Pop M, Salzberg SL (2009). Ultrafast and memory-efficient alignment of short DNA sequences to the human genome. Genome Biol..

[59] Clark SJ, Statham A, Stirzaker C, Molloy PL, Frommer M (2006). DNA methylation: bisulphite modification and analysis. Nat. Protoc..

[60] Pidsley R (2016). Critical evaluation of the Illumina MethylationEPIC BeadChip microarray for whole-genome DNA methylation profiling. Genome Biol..

[61] Song Q (2013). A reference methylome database and analysis pipeline to facilitate integrative and comparative epigenomics. PLoS One.

[62] Robinson MD, McCarthy DJ, Smyth GK (2010). edgeR: a Bioconductor package for differential expression analysis of digital gene expression data. Bioinformatics.

[63] Taberlay PC, Statham AL, Kelly TK, Clark SJ, Jones PA (2014). Reconfiguration of nucleosome-depleted regions at distal regulatory elements accompanies DNA methylation of enhancers and insulators in cancer. Genome Res..

[64] Valdes-Mora F (2017). Acetylated histone variant H2A.Z is involved in the activation of neo-enhancers in prostate cancer. Nat. Commun..

[65] Hattori T (2013). Recombinant antibodies to histone post-translational modifications. Nat. Methods.

[66] Lund EG, Duband-Goulet I, Oldenburg A, Buendia B, Collas P (2015). Distinct features of lamin A-interacting chromatin domains mapped by ChIP-sequencing from sonicated or micrococcal nuclease-digested chromatin. Nucleus.

[67] Feng, X., Grossman, R. & Stein, L. PeakRanger: a cloud-enabled peak caller for ChIP-seq data. *BMC Bioinformatics* **12**, 139 (2011). 10.1186/1471-2105-12-139 PMC3103446 21554709

[68] Lund E, Oldenburg AR, Collas P (2014). Enriched domain detector: a program for detection of wide genomic enrichment domains robust against local variations. Nucleic Acids Res..

[69] Gardiner-Garden, M. & Frommer, M. CpG islands in vertebrate genomes. *J. Mol. Biol.* **196**, 261–282 (1987). 10.1016/0022-2836(87)90689-9 3656447

[70] Lawrence M (2013). Software for computing and annotating genomic ranges. PLoS Comput. Biol..

[71] Sheffield NC, Bock C (2016). LOLA: enrichment analysis for genomic region sets and regulatory elements in R and Bioconductor. Bioinformatics.

[72] Akalin A, Franke V, Vlahovicek K, Mason CE, Schubeler D (2015). Genomation: a toolkit to summarize, annotate and visualize genomic intervals. Bioinformatics.

[73] Wickham, H. *ggplot2: Elegant Graphics for Data Analysis* (Springer, 2016).

[74] Ernst J, Kellis M (2012). ChromHMM: automating chromatin-state discovery and characterization. Nat. Methods.

[75] Ritchie ME (2015). limma powers differential expression analyses for RNA-sequencing and microarray studies. Nucleic Acids Res..

[76] Subramanian A (2005). Gene set enrichment analysis: a knowledge-based approach for interpreting genome-wide expression profiles. Proc. Natl. Acad. Sci. U.S.A..

[77] Liberzon A (2015). The molecular signatures database hallmark gene set collection. Cell Syst..

[78] Bickmore WA (2013). The spatial organization of the human genome. Annu. Rev. Genomics Hum. Genet..

[79] Gilbert DM (2002). Replication timing and transcriptional control: beyond cause and effect. Curr. Opin. Cell Biol..

