# Supplementary material for: Replication timing and epigenome remodelling are associated with the nature of chromosomal rearrangements in cancer
Source: Nat Commun. 2019 Jan 24;10:416. doi: 10.1038/s41467-019-08302-1 (PMC6345877; doi:10.1038/s41467-019-08302-1)
Supplement: Supplementary file 1 — Supplementary Information [file 41467_2019_8302_MOESM1_ESM.pdf]

**Replication timing and epigenome remodelling are associated with the nature of chromosomal rearrangements in cancer**

**Du et al.**

- **Supplementary Figures 1 – 14**
- **Supplementary Tables 1 – 6**

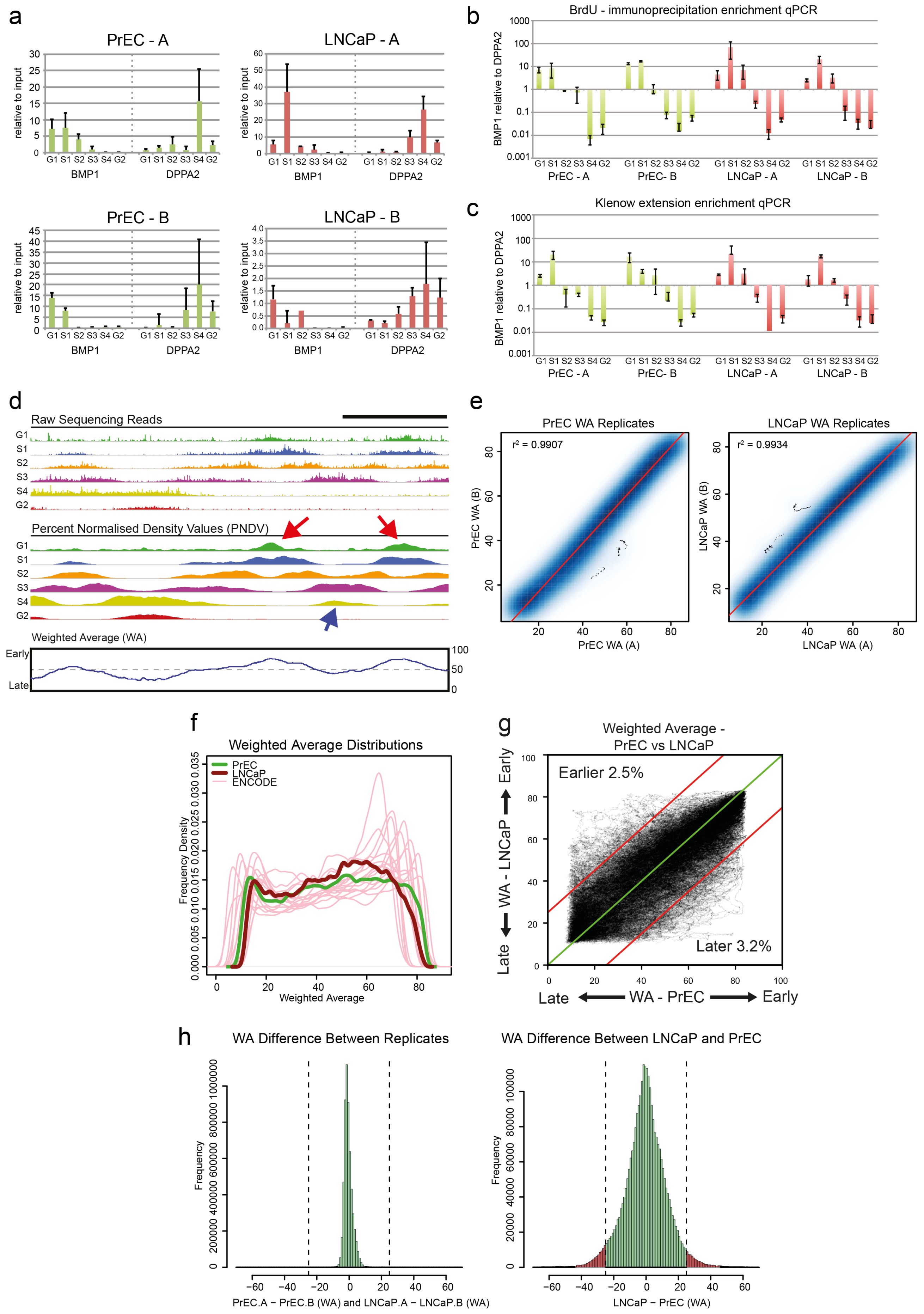


**Supplementary Figure 1: Validation of Repli-Seq datasets and replication timing difference thresholds.**

**a** Validation of correct S-phase sorting. qPCR of known early (*BMP1*) and known late (*DPPA2*) loci was carried out on BrdU-labelled DNA extracted from sorted PrEC and LNCaP samples. **b** Relative levels of *BMP1* to *DPPA2* loci were used as a measure of early to late replication. The y-axis uses a $\log_{10}$ scale. **c** qPCR was repeated on the same samples following Klenow dsDNA synthesis. For **a**-**c**, error bars indicate standard deviation of n=3. **d** Raw read densities across all S-phase fractions are normalised to give a Percent Normalised Density Values (PNDV) per 1kb locus for each fraction. Red arrows indicate regions of replication initiation and the blue arrow indicates a region of replication termination. PNDV values of all 6 fractions are used to calculate a single Weighted Average (WA) score per locus. Higher WA values indicate early replication timing and lower WA values indicate late replication timing. Scale bar indicates 1 Mb. **e** Replicates of each cell line show high correlation and $r^2$ scores. **f** The distribution of PrEC (green) and LNCaP (red) WA values are comparable to the WA distributions of twelve ENCODE Repli-Seq datasets (pink). **g** Replication timing weighted average (WA) values for all mappable 1kb loci are compared between PrEC and LNCaP. The green line indicates loci of no change in replication timing between PrEC and LNCaP. Loci outside the red lines ($|\Delta WA| > 25$) are substantially changed in replication timing. **h** The distribution of WA differences between replicates (left) is compared to the distribution of WA differences between PrEC and LNCaP (right). Dotted lines indicate a $|\Delta WA| > 25$. Values that fall outside this range (red) signify loci that have changed replication timing.

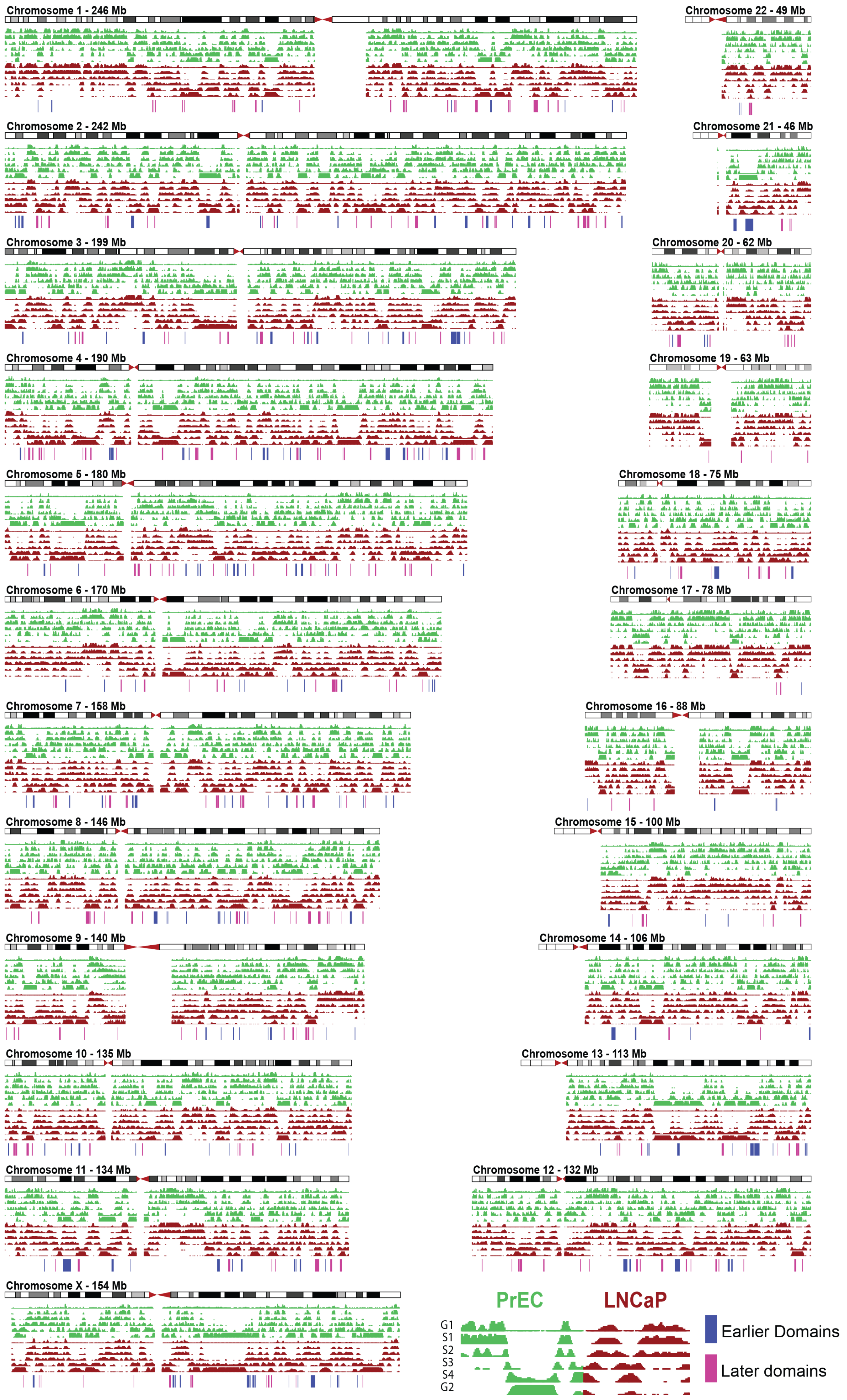
Chromosome 1 - 246 Mb
Chromosome 22 - 49 Mb
Chromosome 2 - 242 Mb
Chromosome 21 - 46 Mb
Chromosome 3 - 199 Mb
Chromosome 20 - 62 Mb
Chromosome 4 - 190 Mb
Chromosome 19 - 63 Mb
Chromosome 5 - 180 Mb
Chromosome 18 - 75 Mb
Chromosome 6 - 170 Mb
Chromosome 17 - 78 Mb
Chromosome 7 - 158 Mb
Chromosome 16 - 88 Mb
Chromosome 8 - 146 Mb
Chromosome 15 - 100 Mb
Chromosome 9 - 140 Mb
Chromosome 14 - 106 Mb
Chromosome 10 - 135 Mb
Chromosome 13 - 113 Mb
Chromosome 11 - 134 Mb
Chromosome 12 - 132 Mb
Chromosome X - 154 Mb
PrEC
LNCaP
G1
S1
S2
S3
S4
G2
Earlier Domains
Later domains

**Supplementary Figure 2: Replication timing in the normal and cancer prostate genome.**
Repli-Seq derived PNDV values of all cell fractions are plotted for each chromosome (hg18). The name and size of each chromosome is shown above the respective chromosomal ideogram. Green values represent PrEC and red values represent LNCaP. Domains that replicate *earlier* in LNCaP are highlighted in blue; domains that replicate *later* in LNCaP are highlighted in magenta. An efficient replication origin cluster is shaped as an inverted V in the PNDV signal, indicative of a bidirectional replication fork progressing from early in S-phase (tip of inverted V) towards late (bottom of inverted V)[1,2]. Notably, *later* and *earlier* domains are also located in regions of inverted V shapes for both PrEC or LNCaP cells, suggesting that these domains are associated with a change in replication origin firing.

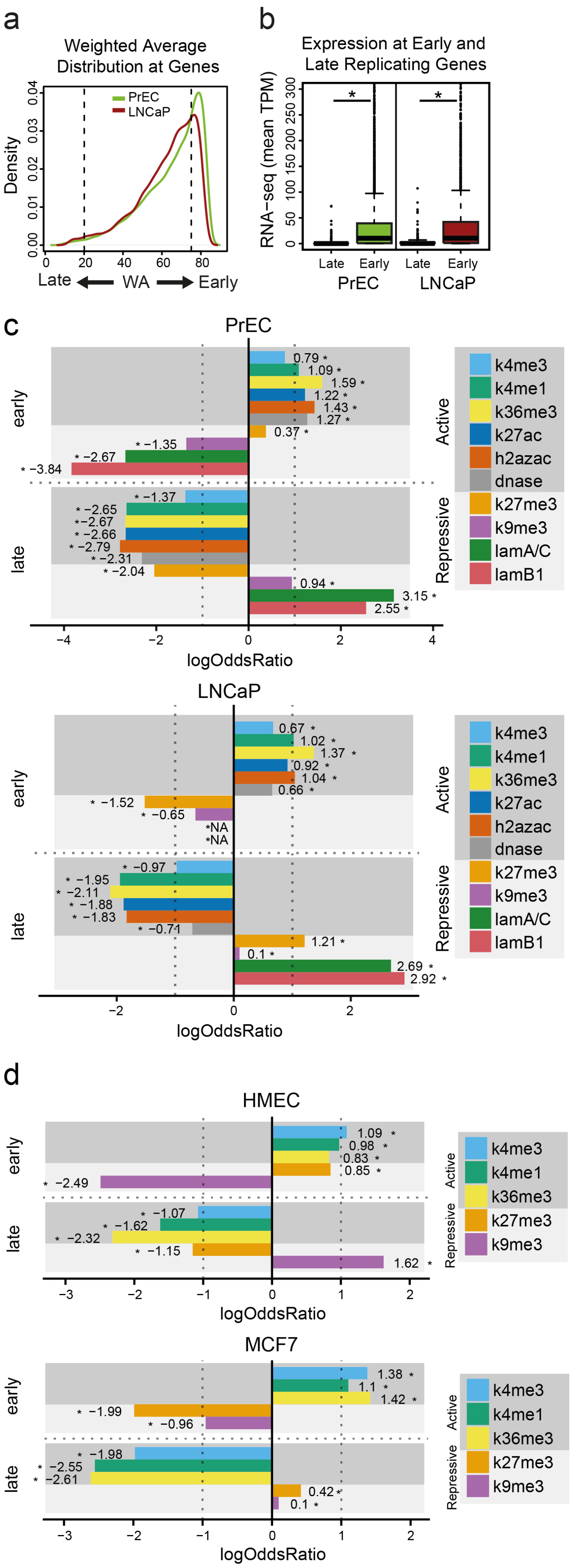


**Supplementary Figure 3: Relationship between gene expression, chromatin marks and replication timing.**
**a** The density of WA scores at gene promoters for PrEC and LNCaP. Genes are *earlier* replicating compared to the whole genome for both PrEC and LNCaP ($p < 2.2e-16$, one-tailed Mann-Whitney-Wilcoxon). **b** Expression levels for genes that replicate early (WA > 75) and late (WA < 20) in PrEC and LNCaP. Early-replicating genes have higher expression than late-replicating genes (asterisks, $p < 2.2e-16$, one-tailed Mann-Whitney-Wilcoxon). For boxplots, centerline indicates the median, box limits indicate upper and lower quartiles, whiskers indicate the 1.5 interquartile range and points indicate outliers. **c** Associations between chromatin marks, and early and late replication timing in PrEC and LNCaP. **d** Associations between chromatin marks in HMEC and MCF7, and early and late replication timing in MCF7. For **c** and **d**, association is above zero, and disassociation is below zero. Asterisks indicate significant associations (FDR < 0.05, Fisher's exact test).

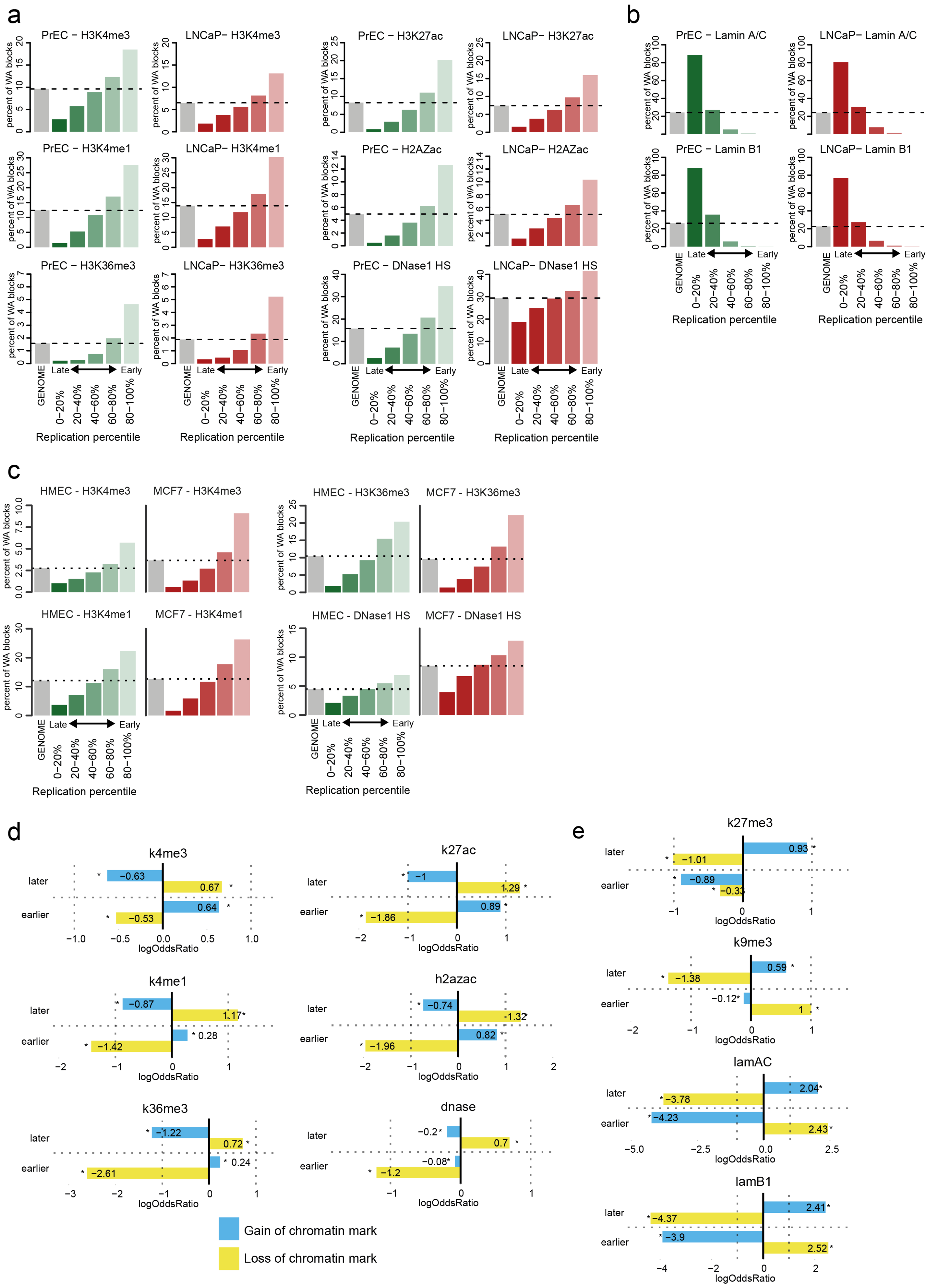
a
PrEC – H3K4me3
LNCaP– H3K4me3
PrEC – H3K27ac
LNCaP– H3K27ac
PrEC – H3K4me1
LNCaP– H3K4me1
PrEC – H2AZac
LNCaP– H2AZac
PrEC – H3K36me3
LNCaP– H3K36me3
PrEC – DNase1 HS
LNCaP– DNase1 HS
percent of WA blocks
Late
Early
GENOME
0−20%
20−40%
40−60%
60−80%
80−100%
Replication percentile
b
PrEC – Lamin A/C
LNCaP– Lamin A/C
PrEC – Lamin B1
LNCaP– Lamin B1
c
HMEC - H3K4me3
MCF7 - H3K4me3
HMEC - H3K36me3
MCF7 - H3K36me3
HMEC - H3K4me1
MCF7 - H3K4me1
HMEC - DNase1 HS
MCF7 - DNase1 HS
d
k4me3
k27ac
k4me1
h2azac
k36me3
dnase
later
earlier
logOddsRatio
−0.63
0.67
0.64
−0.53
−1
1.29
0.89
−1.86
−0.87
1.17
0.28
−1.42
−0.74
1.32
0.82
−1.96
−1.22
0.72
0.24
−2.61
−0.2
0.7
−0.08
−1.2
Gain of chromatin mark
Loss of chromatin mark
e
k27me3
k9me3
lamAC
lamB1
0.93
−1.01
−0.89
−0.33
0.59
−1.38
−0.12
1
2.04
−3.78
−4.23
2.43
2.41
−4.37
−3.9
2.52

**Supplementary Figure 4: The distribution of chromatin marks across replication timing and associations with replication timing alterations.**

Percentage occupancy of chromatin marks for 1kb loci across replication timing for active **a** and repressive **b** marks in PrEC and LNCaP. Each mark is compared against the percentage of genome occupancy for that mark. The same was done for HMEC and MCF7 using MCF7 replication timing **c**. Associations between loci that change in replication timing and loci that change in active **d** or repressive **e** chromatin mark occupancy between PrEC and LNCaP. Positive values (RHS) indicate association between the difference in chromatin mark occupancy and the change in timing. Negative values (LHS) indicated disassociation between the difference in chromatin mark occupancy and the change in timing. Asterisks indicate significant associations (FDR < 0.05, Fisher's exact test).

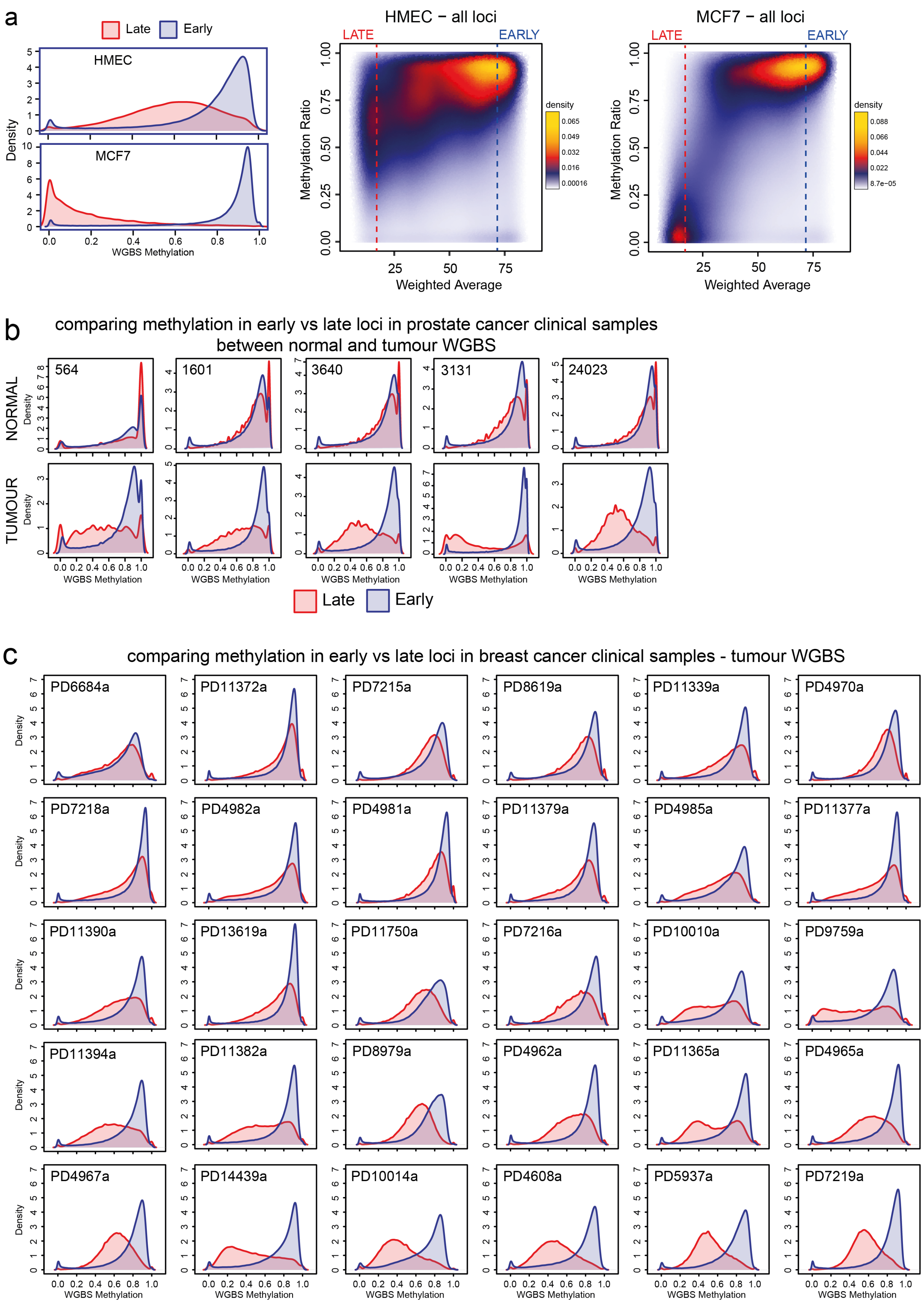
a
Late
Early
HMEC
MCF7
Density
WGBS Methylation
HMEC − all loci
LATE
EARLY
Methylation Ratio
Weighted Average
density
0.065
0.049
0.032
0.016
0.00016
MCF7 − all loci
0.088
0.066
0.044
0.022
8.7e−05
b
comparing methylation in early vs late loci in prostate cancer clinical samples
between normal and tumour WGBS
NORMAL
TUMOUR
564
1601
3640
3131
24023
c
comparing methylation in early vs late loci in breast cancer clinical samples - tumour WGBS
PD6684a
PD11372a
PD7215a
PD8619a
PD11339a
PD4970a
PD7218a
PD4982a
PD4981a
PD11379a
PD4985a
PD11377a
PD11390a
PD13619a
PD11750a
PD7216a
PD10010a
PD9759a
PD11394a
PD11382a
PD8979a
PD4962a
PD11365a
PD4965a
PD4967a
PD14439a
PD10014a
PD4608a
PD5937a
PD7219a

**Supplementary Figure 5: Replication timing correlates with DNA methylation in cancer.**
**a** DNA methylation (WGBS) density distributions for early (blue) and late (red) loci for HMEC and MCF7. Adjacent are scatterplots of DNA methylation in relation to replication timing (WA) for all measured 1kb loci in HMEC and MCF7. Blue dashed line indicates early (WA > 73) and red dashed line indicates late (WA < 17). **b** DNA methylation density distributions for early and late loci in 5 paired clinical prostate cancer patient samples (GSE104789). PrEC WA scores were used to stratify loci for 'Normal' into early or late and LNCaP WA scores were used to stratify loci for 'Tumour' into early or late. **c** DNA methylation density distributions for early and late loci in 30 public breast cancer patient WGBS samples from Brinkman *et al.* (2018)[3]. Only CpG sites with coverage of at least 4 reads were included. MCF7 WA scores were used to stratify loci into early or late. Breast cancer patient WGBS datasets (bigwigs) from Brinkman *et al.* (2018) (biorxiv) were downloaded from https://zenodo.org/record/1217427#.W1gB1SN7GFA.

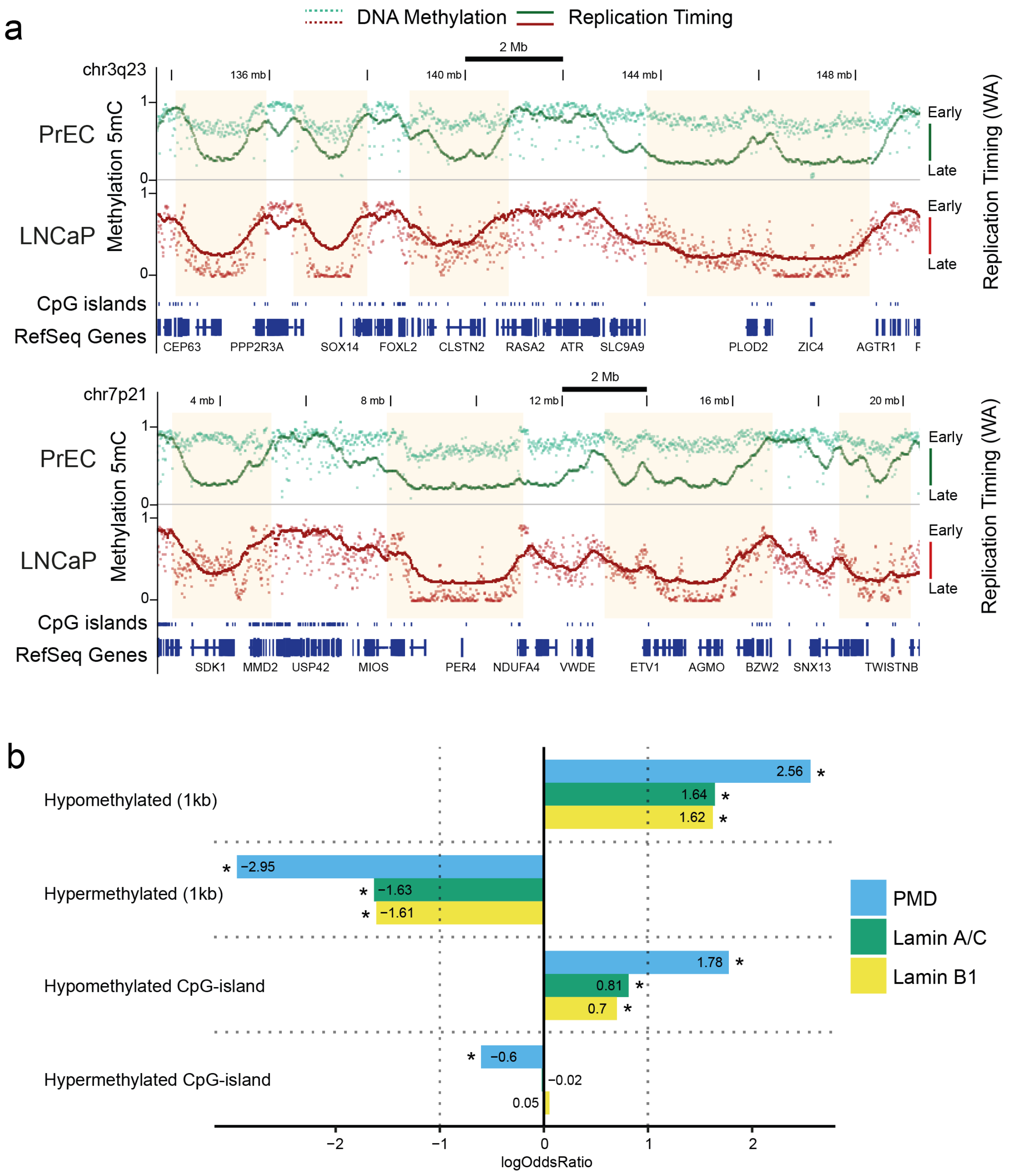


**Supplementary Figure 6: Relationship between replication timing and DNA methylation.**
**a** More representative examples of late-replicating regions in both PrEC and LNCaP (shaded) that become hypomethylated in LNCaP (see Fig. 2b). Scale bar represents 2 Mb. **b** Associations between LNCaP PMDs and LADs, and hypo- and hypermethylated 1kb loci and CpG-islands (LNCaP-PrEC, $\Delta$WGBS > |0.2|). Association is above zero, and disassociation is below zero. Asterisks indicate significant associations (FDR < 0.05, Fisher's exact test). We observe no association of hypermethylated loci or hypermethylated CpG-islands with PMDs and LADs. Our result contrasts with Berman *et al.* (2012)[4] who reported that in colorectal cancer, hypermethylated CpG-islands were primarily found within partially methylated domains (PMDs), coinciding with late-replicating fibroblast LADs. The findings of variable CpG-island hypermethylation patterns could potentially relate to differences in the tissue-of-origin[5], differences in LADs from different cell-of-origins, or the different CpG-island methylation phenotypes between prostate and colon cancer.

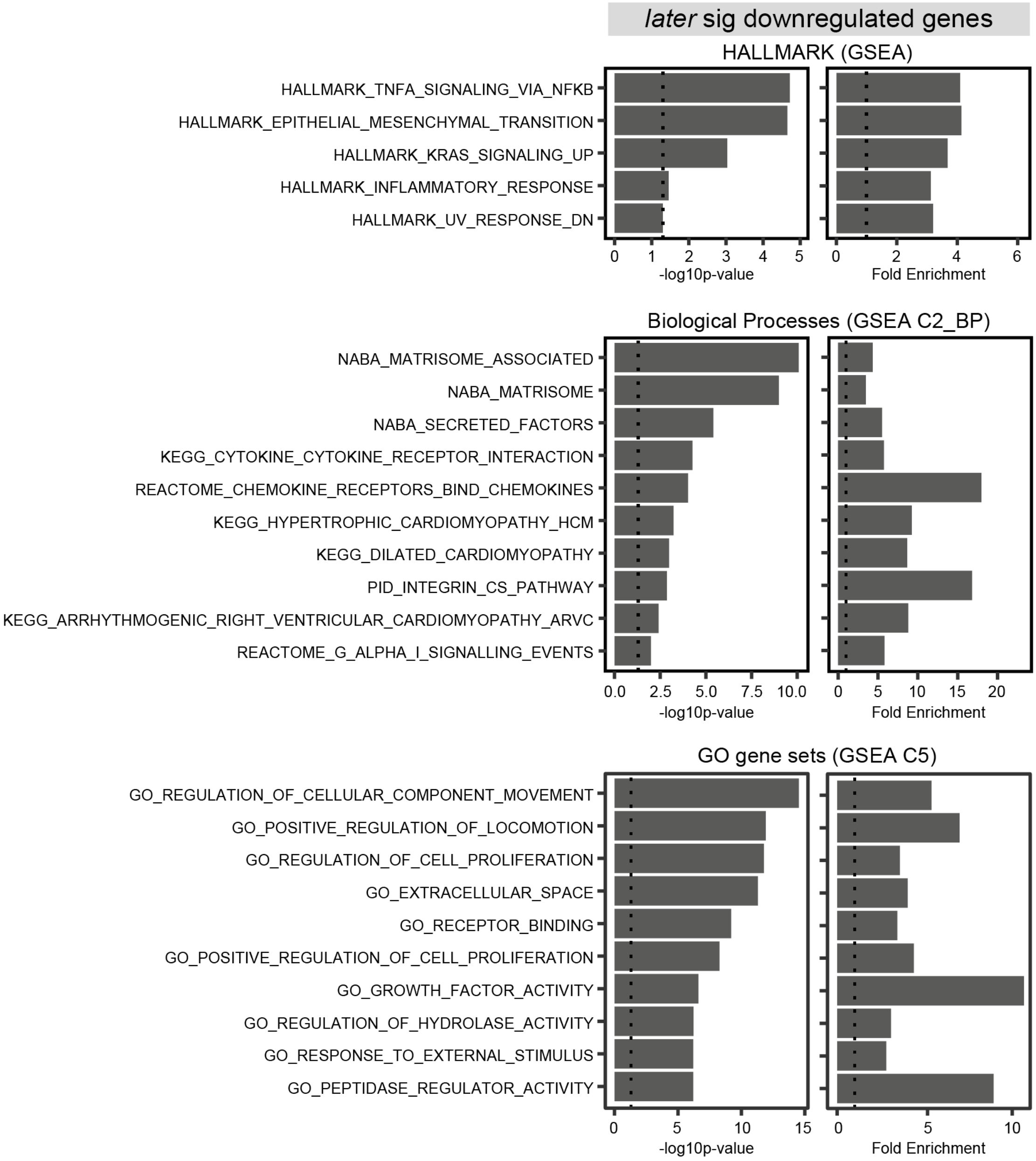


**Supplementary Figure 7: Significant GSEA terms for genes found within *later* domains.**
Genes within *later* domains that were also downregulated (FDR < 0.01, LNCaP/PrEC logFC < -1.5) were tested against GSEA MolSigDB v6.0 gene sets. We used a hyper-geometric test to identify statistically significant enrichment (p < 0.05, bonferroni corrected). Only gene sets with significant terms are shown. If there are more than 10 significant terms, only the top 10 are shown. The left hand side plot shows the significant terms ranked by -log10(p-value). Dotted line on the -log10(p-value) axis is the significance cutoff. The right hand side plot shows the fold enrichment for each term. Dotted line on the fold enrichment axis indicates where fold enrichment =1.

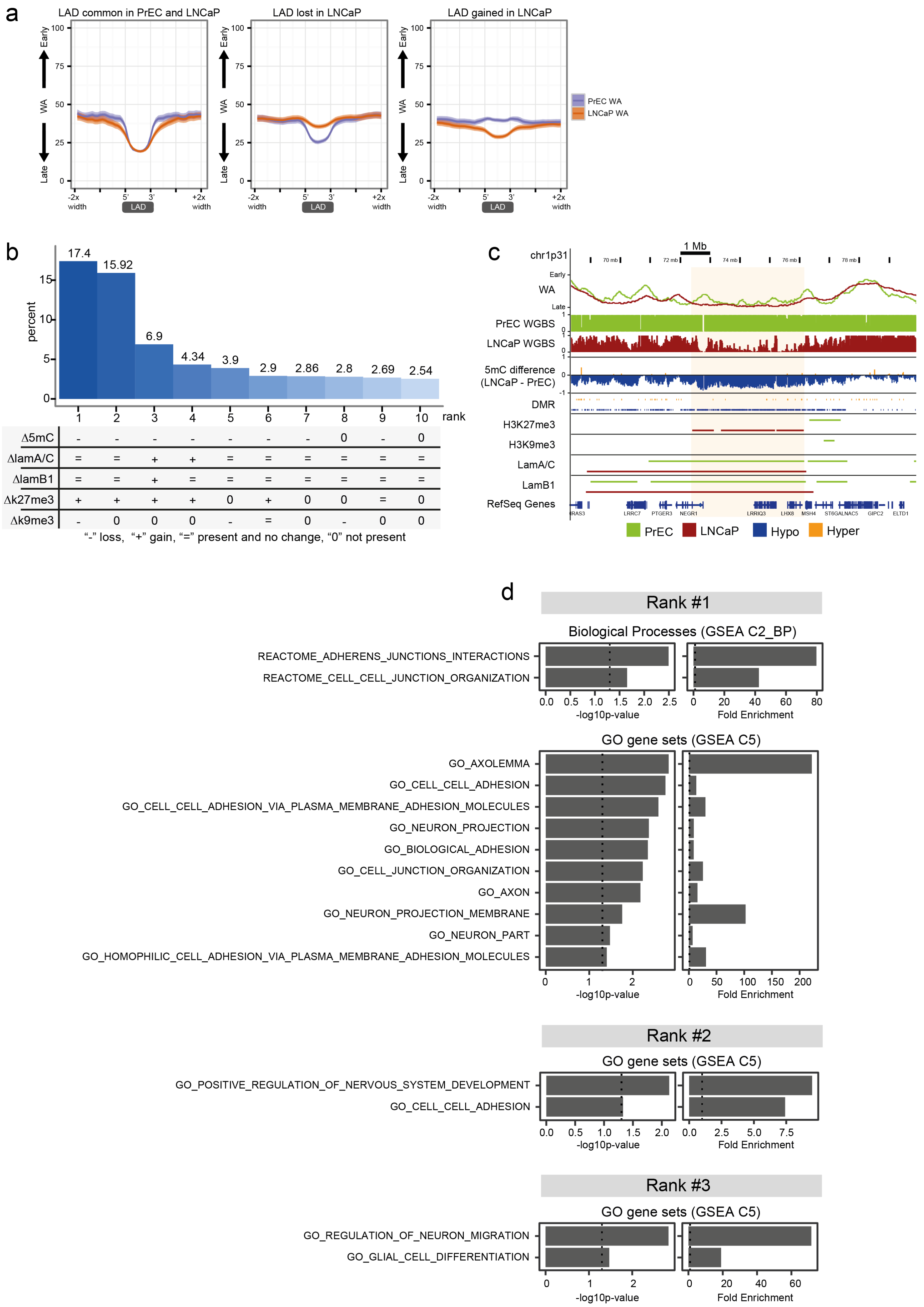
a
LAD common in PrEC and LNCaP
LAD lost in LNCaP
LAD gained in LNCaP
Early
WA
Late
100
75
50
25
0
-2x width
5'
3'
+2x width
LAD
PrEC WA
LNCaP WA
b
percent
17.4
15.92
6.9
4.34
3.9
2.9
2.86
2.8
2.69
2.54
rank
1 2 3 4 5 6 7 8 9 10
Δ5mC - - - - - - - 0 - 0
ΔlamA/C = = + + = = = = = =
ΔlamB1 = = + = = = = = = =
Δk27me3 + + + + 0 + 0 0 = 0
Δk9me3 - 0 0 0 - = 0 - 0 0
"-" loss, "+" gain, "=" present and no change, "0" not present
c
chr1p31
1 Mb
70 mb
72 mb
74 mb
76 mb
78 mb
WA
PrEC WGBS
LNCaP WGBS
5mC difference (LNCaP - PrEC)
DMR
H3K27me3
H3K9me3
LamA/C
LamB1
RefSeq Genes
LRRC7
PTGER3
NEGR1
LRRIQ3
LHX8
MSH4
ST6GALNAC5
GIPC2
ELTD1
PrEC
LNCaP
Hypo
Hyper
d
Rank #1
Biological Processes (GSEA C2_BP)
REACTOME_ADHERENS_JUNCTIONS_INTERACTIONS
REACTOME_CELL_CELL_JUNCTION_ORGANIZATION
GO gene sets (GSEA C5)
GO_AXOLEMMA
GO_CELL_CELL_ADHESION
GO_CELL_CELL_ADHESION_VIA_PLASMA_MEMBRANE_ADHESION_MOLECULES
GO_NEURON_PROJECTION
GO_BIOLOGICAL_ADHESION
GO_CELL_JUNCTION_ORGANIZATION
GO_AXON
GO_NEURON_PROJECTION_MEMBRANE
GO_NEURON_PART
GO_HOMOPHILIC_CELL_ADHESION_VIA_PLASMA_MEMBRANE_ADHESION_MOLECULES
Rank #2
GO gene sets (GSEA C5)
GO_POSITIVE_REGULATION_OF_NERVOUS_SYSTEM_DEVELOPMENT
GO_CELL_CELL_ADHESION
Rank #3
GO gene sets (GSEA C5)
GO_REGULATION_OF_NEURON_MIGRATION
GO_GLIAL_CELL_DIFFERENTIATION
-log10p-value
Fold Enrichment

**Supplementary Figure 8: Long-range epigenetically regulated domains in cancer change in replication timing.**

**a** Average plots of PrEC and LNCaP WA values over regions containing both lamin A/C and B1 LADs, that are conserved between PrEC and LNCaP, lost in LNCaP or gained in LNCaP. LADs lost in LNCaP are significantly *earlier* in LNCaP than PrEC ($p < 2.2e-16$, one-tailed Mann-Whitney-Wilcoxon), and LADs gained in LNCaP are significantly *later* in LNCaP than PrEC ($p < 2.2e-16$, one-tailed Mann-Whitney-Wilcoxon). Plots show an average line with width of shading indicating confidence intervals. **b** The top ten most common combinations of changes in DNA methylation, lamina association, and heterochromatin within LNCaP late-replicating loci. The table below illustrates which combination of changes has occurred. Comparing LNCaP to PrEC, "-" indicates loss of that mark, "+" indicates gain of that mark, "=" indicates that the mark is present and not changed, and "0" indicates that the mark is not present in either cell line. **c** A representative example of a late-replicating region in LNCaP showing maintained LADs with coordinate DNA hypomethylation and H3K27me3 gain, without presence of H3K9me3. **d** Genes within rank 1, 2 and 3 regions (see **b**) were tested against GSEA MolSigDB v6.0 gene sets. We used a hyper-geometric test to identify statistically significant enrichment ($p < 0.05$, bonferroni corrected). Only gene sets with significant terms are shown. If there are more than 10 significant terms, only the top 10 are shown. The left hand side plot shows the significant terms ranked by -log10(p-value). Dotted line on the -log10(p-value) axis is the significance cutoff. The right hand side plot shows the fold enrichment for each term. Dotted line on the fold enrichment axis indicates where fold enrichment =1.

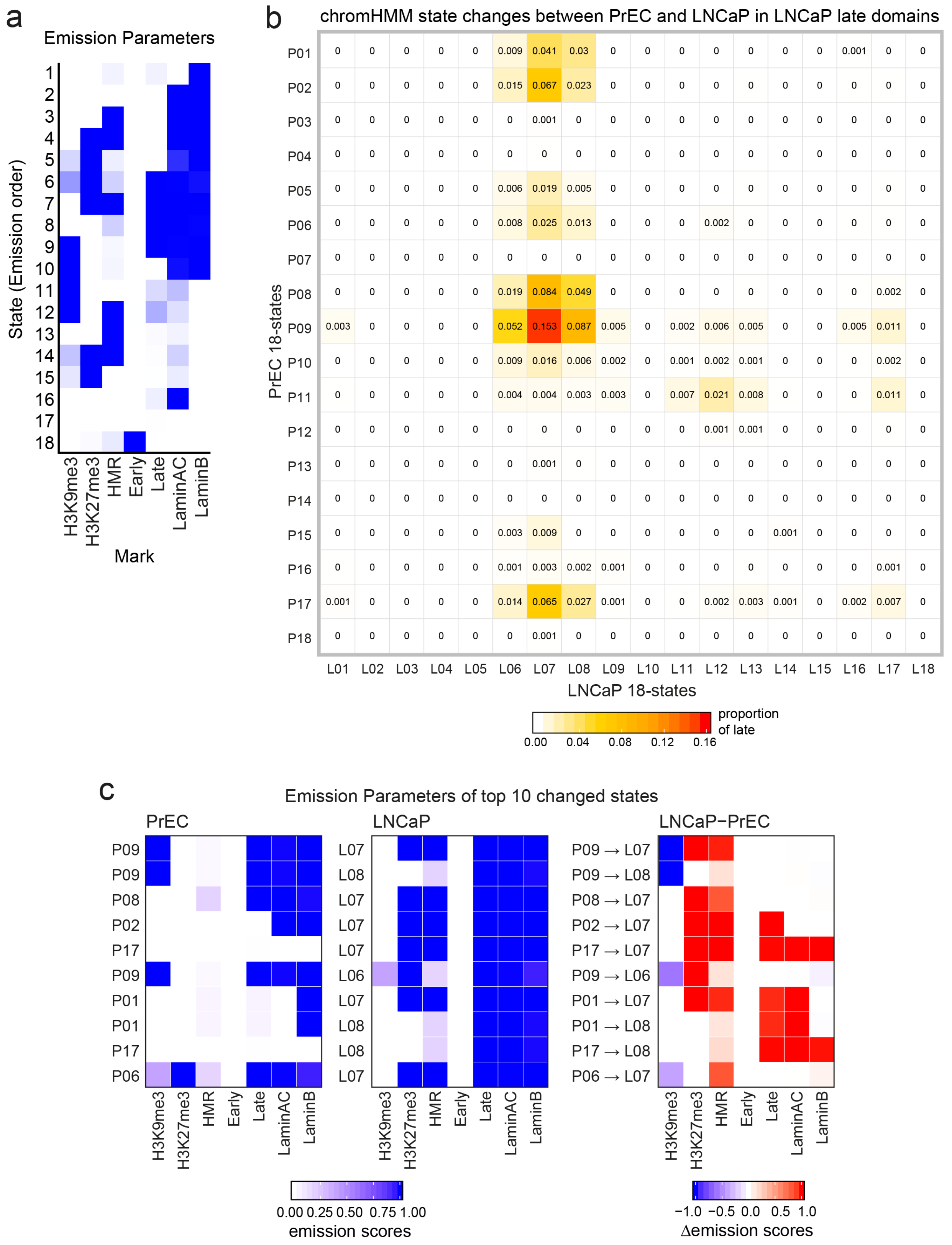


**Supplementary Figure 9: DNA hypomethylation and heterochromatin alterations in cancer occur concordantly with in late replicating LADs.**
**a** Emission parameters (mark probabilities) of the 18-state ChromHMM model learnt on PrEC and LNCaP data. Marks are H3K27me3, H3K9me3, Hypomethylated Regions, Early, Late, lamin A/C LAD, lamin B1 LAD. **b** Proportions of pairwise state changes in LNCaP late-replicating loci. **c** Emission parameters of the top 10 states changes between PrEC and LNCaP, ranked from highest to lowest proportion. The emission intensities indicate the probability of the histone mark to be found in that state. The 'Δemission scores' heatmap indicate which marks have changed between the paired states.

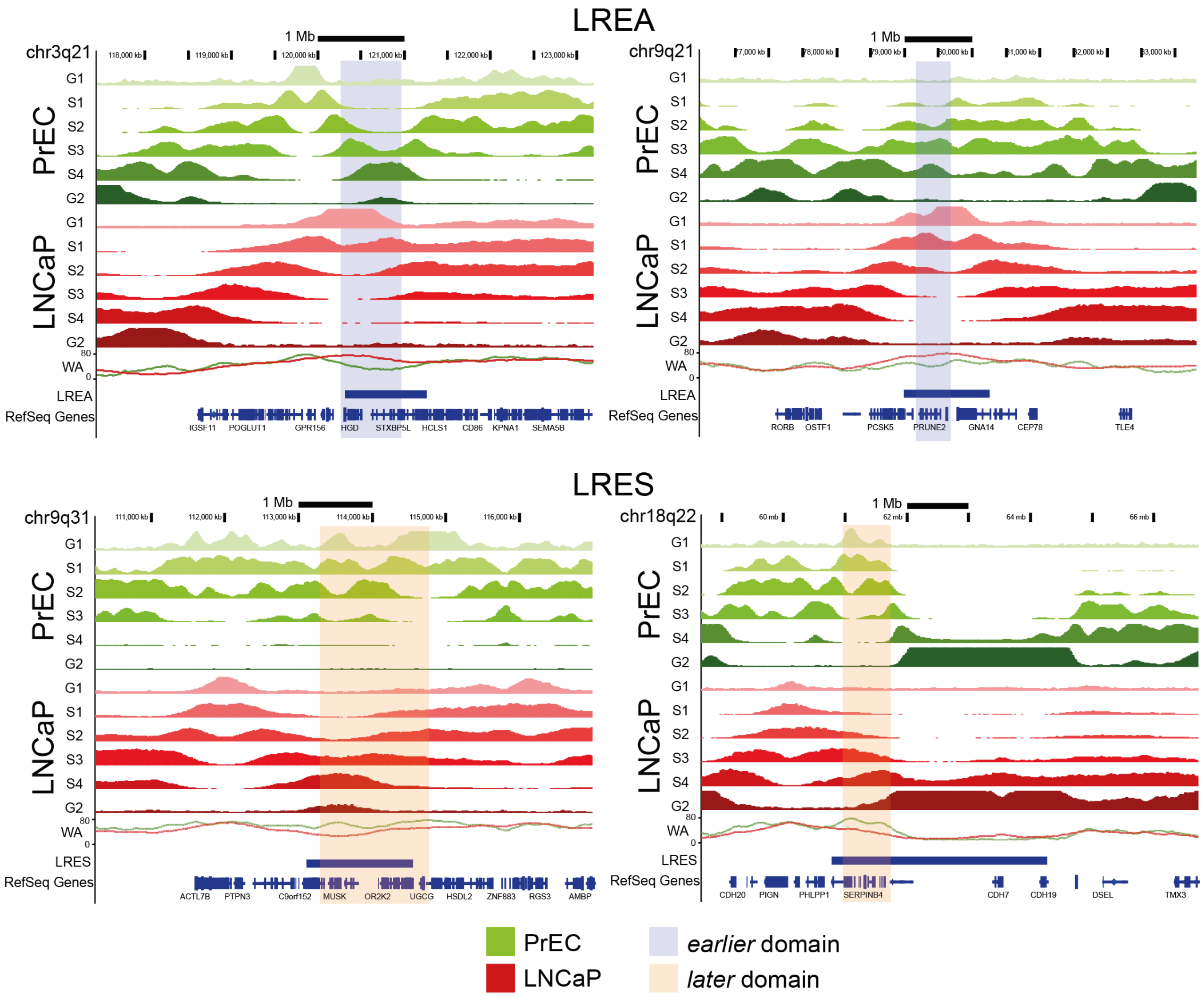


**Supplementary Figure 10: Examples of domains of replication timing change overlapping LRES and LREA regions.**

PrEC and LNCaP data are respectively represented by the colors green and red. Blue shading indicates an *earlier* domain. Pink shading indicates a *later* domain.

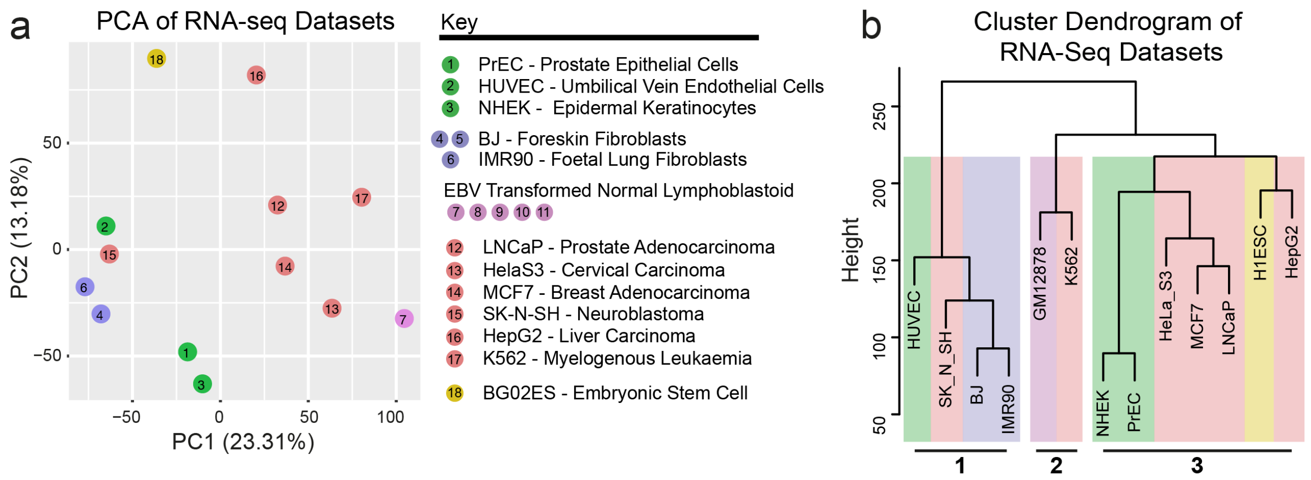


**Supplementary Figure 11: RNA-seq data does not clearly separate cancer and normal cell lines.** Replicate averaged logCPM values from the ENCODE RNA-seq datasets, including PrEC and LNCaP, are assessed using PCA **a** and hierarchical clustering (Ward's criterion) **b**. Clusterwise Jaccard bootstrap means are 0.7793 (cluster 1), 0.6666 (cluster 2) and 0.6834 (cluster 3). Mean values for clusters 2 and 3 are below 0.8, indicating unstable clusters. Samples are identifiable by colour and number key.

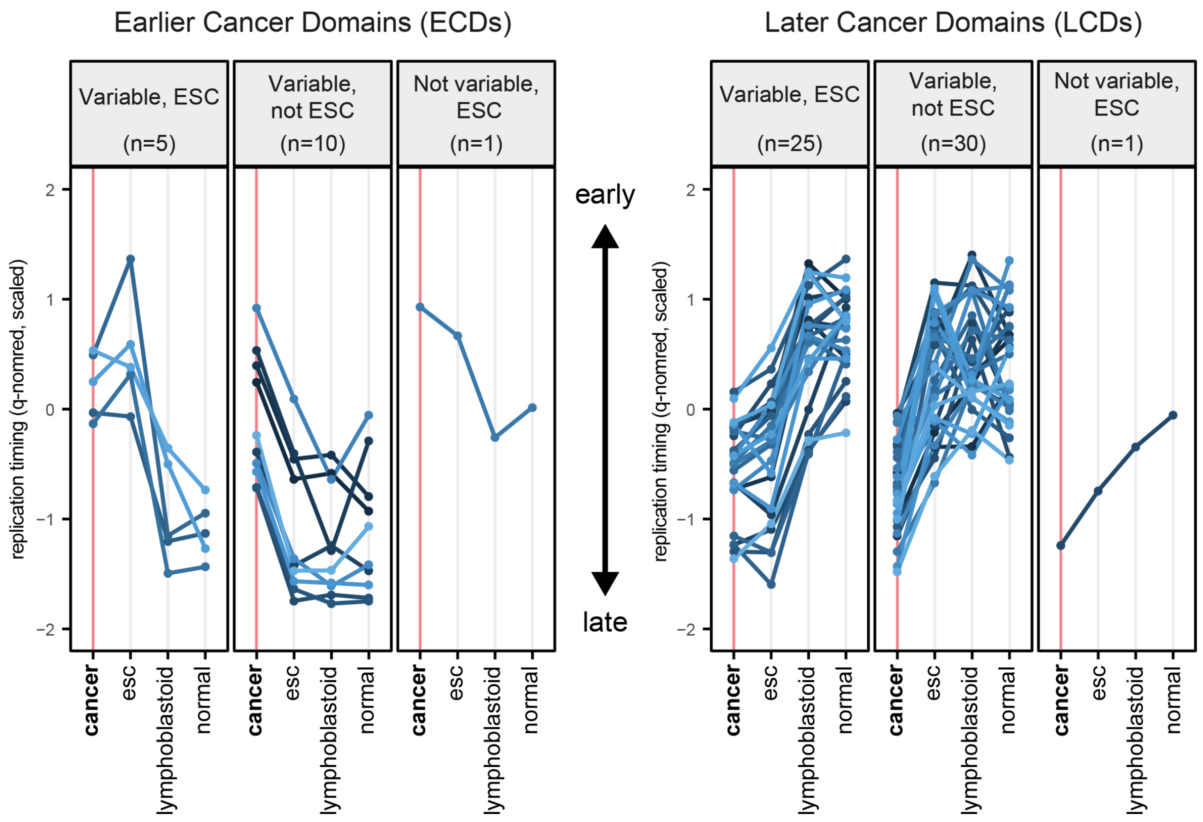


**Supplementary Figure 12: Replication timing of cancer compared to other cell types in Earlier Cancer Domains and Later Cancer Domains.**

Averaged replication timing scores for cancer (HEPG2, K562, MCF7, SKNSH, LNCaP, HELAS3), embryonic stem cell (BG02-ESC), lymphoblastoids (GM06990, GM12801, GM12812, GM12813, GM12878) and normal (BJ_Rep1, BJ_Rep2, IMR90, HUVEC, NHEK, PrEC) across ECDs and

LCDs are represented by the dot along each vertical parallel coordinate for that cell group. Each line represents a single domain and connects the averaged scores (dots) of the 4 cell groups for that domain. 'Variable' indicates domains that overlap with regions of high variation between cell types (See Methods). 'ESC' indicates domains where cancer replication timing corresponds to ESC replication timing (See Methods). The majority of ECDs and LCDs overlap regions of high variation between cell types. Moreover, a large subset of ECDs (10/16) and LCDs (30/56) are differentially timed between cancer and ESCs, indicating that some *earlier* replicating and *later* replicating domains are cancer specific. ECDs show consistently higher replication timing scores in cancer compared to lymphoblastoid and normal, and higher than ESC in domains that do not associate with ESC timing ('Variable, not ESC'). LCDs show consistently lower replication timing scores in cancer compared to lymphoblastoid and normal, and lower than ESCs in domains that do not associate with ESC timing ('Variable, not ESC'). The timing scores (WA) shown here are after quantile-normalisation and scaling (See Methods).

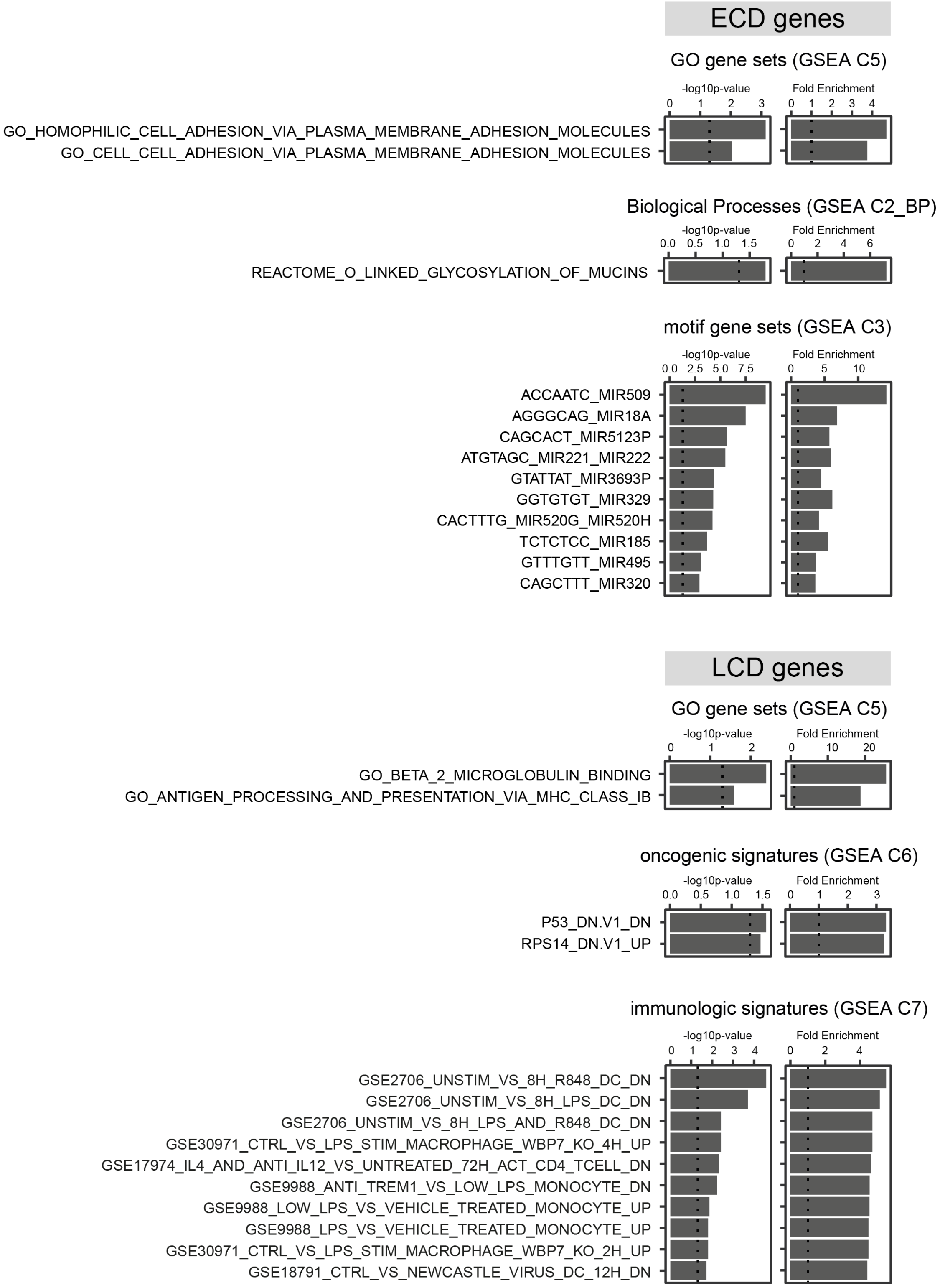


**Supplementary Figure 13: Significant GSEA terms for genes found within ECDs or LCDs.** ECD and LCD genes from domains called with a logFC > 0 cutoff were tested against GSEA MolSigDB v6.0 gene sets. We used a hyper-geometric test to identify statistically significant enrichment ($p < 0.05$, bonferroni corrected) of gene sets with ECD and LCD genes. Only gene sets with significant terms are shown here. If there are more than 10 significant terms, only the top 10 are shown. The left hand side plot shows the significant terms ranked by -log10(p-value). Dotted line on the -log10(p-value) axis is the significance cutoff. The right hand side plot shows the fold enrichment for each term. Dotted line on the fold enrichment axis indicates where fold enrichment =1.

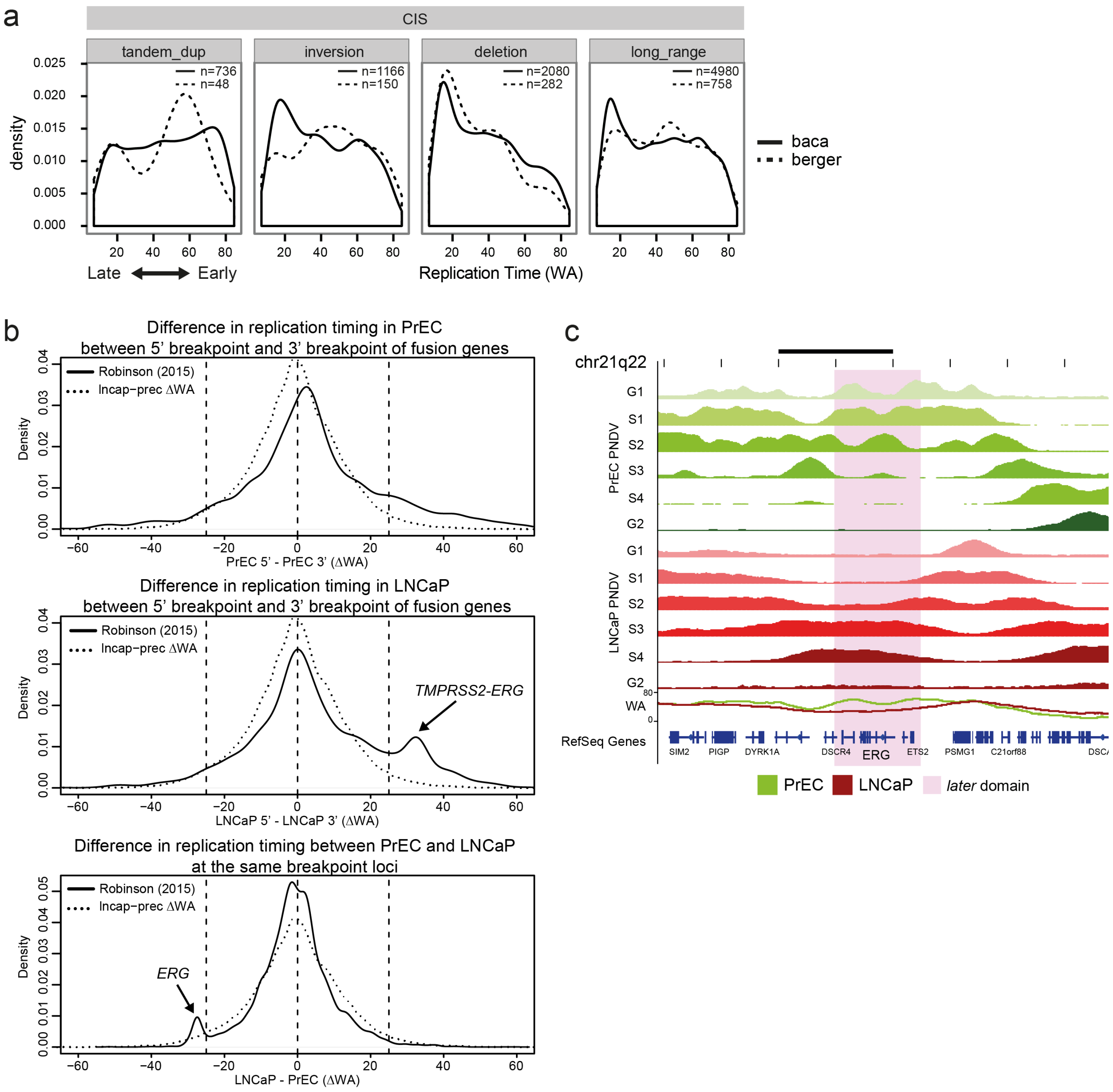


**Supplementary Figure 14: Relationship between replication timing and chromosomal rearrangements.**
**a** Replication timing (PrEC WA) distributions for chromosomal rearrangements divided into tandem duplications, inversions, deletions and long-range cis rearrangements. Solid lines are distributions for Baca *et. al.* (2013) and dashed lines are distributions for Berger *et. al.* (2011). **b** Replication timing differences within PrEC and LNCaP (top and middle panel) between the 5' and the 3' gene fusion breakpoints described from clinical prostate cancer samples[6], and replication timing differences between PrEC and LNCaP of the same breakpoint (bottom panel). Distributions of breakpoint timing differences are compared to genome wide timing differences between PrEC and LNCaP (dotted line). **c** Replication timing change across the *ERG* locus. Scale bar represents 1 Mb.

| | |
|---|---|
| ***earlier*** **upregulated** | AC018865.8, AC079776.7, AC092667.2, AGXT, ARG1, ASZ1, ATP8A1, BAMBI, CACNA2D3, CARTPT, CCDC67, CHPT1, CMPK2, COBL, CTD-2251F13.1, CYP7A1, DDIT4L, EEF1DP3, ENPP3, FAM110B, FOLH1B, GFPT2, GPC6, GUCY1A3, GUCY1B2, GUCY1B3, GULP1, HGD, HTR3A, IGF1, IGSF21, JAKMIP1, KCNK5, KIF1A, LIMCH1, LIN7A, LINC00478, LUZP2, MBLAC2, MKRN3, MSMB, MYBPC1, NAALADL2, NCAM2, NDN, NDUFB4, NETO1, NLGN1, PPFIA2, PRKD1, PRUNE2, RNF144A, RP11-17A1.3, RP11-266A24.1, RP11-315E17.1, RP11-466P24.7, RP11-523G9.3, RP11-588K22.2, RP11-676J15.1, RP11-790I12.2, SHISA3, SLC4A4, SMCO4, SPIC, SSTR5, STXBP5L, SVIP, SYT4, TOX3, TRGC1, TRGC2, TRGV9, UGT2B11, UGT2B28, UGT8, ZBED3-AS1, ZBTB16, ZNF385B |
| ***later*** **downregulated** | AC005083.1, AC092614.2, AC098614.2, ADAM19, AGFG1, AKR1B10, AKR1E2, AL162497.1, AMOTL2, ANTXR2, ANXA1, ANXA5, AP000330.8, ARHGAP18, ARL4C, ARRDC4, ARSJ, ASPHD2, BTC, C11orf63, C12orf55, C15orf41, C1orf74, C7orf57, CARD16, CASP1, CCL20, CD1D, CD274, CDK17, CLMP, COL22A1, CTA-211A9.5, CTA-293F17.1, CTA-445C9.15, CTB-11I22.2, CTSC, CXCL1, CXCL2, CXCL3, CXCL5, CXCL6, CYP24A1, DAW1, DCBLD2, DKK1, DLC1, DLGAP1-AS2, EFCAB1, EFEMP1, EPGN, EREG, ETS1, ETS2, EXT1, FAM3C, FAM83B, FAT1, FGF2, FGF5, FGFBP1, FHL2, FRMD6, FST, FYB, FZD10, FZD10-AS1, G0S2, GBP3, GLIPR1, GNG11, HAS2, HAS2-AS1, HMGA2, HMSD, HS3ST1, HSD11B1, IFI16, IFIT5, IFNE, IKZF2, IL21R, IL4R, IL8, IRF6, IRS1, IRS2, ITGA1, ITGA2, ITGAV, ITGB1, ITGB6, ITGB8, IVL, JAG1, KCNJ15, KDSR, KIAA0040, KIRREL, KLF6, KYNU, LAMA2, LAMB3, LINC00460, LINC00519, LINC00702, LINC00704, LINC00964, LINC00973, LPAR1, LRRC8C, MACC1, MB21D2, MDFIC, MIR137HG, MIR205HG, MIR31HG, MYO16, NABP1, NIPAL4, NR3C1, NRG1, NRP1, NT5E, PCNPP3, PDCD1LG2, PDGFC, PDLIM1, PDP1, PHLDA1, PLEKHA2, PLSCR1, PMAIP1, PRDM8, PTGS2, PTHLH, PTPRK, PTPRZ1, RAB38, RAD51AP2, RASAL2, RBMS1, RBMS3-AS3, RGS17, RP1-272L16.1, RP1-28O10.1, RP11-1002K11.1, RP11-114H23.1, RP11-114H23.2, RP11-114H23.3, RP11-115J23.1, RP11-166D19.1, RP11-221N13.3, RP11-222K16.2, RP11-255G12.3, RP11-290L1.3, RP11-30P6.6, RP11-346D6.6, RP11-395N3.1, RP11-395N3.2, RP11-397A16.2, RP11-404O13.1, RP11-417L14.1, RP11-448G15.3, RP11-460N11.3, RP11-462L8.1, RP11-479G22.8, RP11-524H19.2, RP11-568K15.1, RP11-702B10.2, RP11-734I18.1, RP11-820L6.1, RP1L1, RP3-523K23.2, RP4-798P15.3, RP5-1007M22.2, RP5-973M2.2, RPSAP52, RUNX2, SAMD9, SAMD9L, SEMA3A, SEMA3E, SERPINB12, SERPINB13, SERPINB2, SERPINB3, SERPINB4, SERPINB5, SERPINB7, SERPINB8, SERPINE2, SERTAD4-AS1, SFTA1P, SH3RF2, SLC16A2, SLC2A9, SLC8A1, SNAI2, SNCA, SOCS6, SOX7, SPCS3, SPRY2, ST3GAL6, ST3GAL6-AS1, SYT14, TFPI2, TGFB2, TGFBI, TGFBR2, TIPARP, TPBG, TPST2, TRIB2, TSHZ3, TSLP, UBASH3B, UPP1, VEGFC, VGLL3, VPS4B, VSNL1, ZBED2, ZNF572 |

**Supplementary Table 1: List of differentially expressed genes found in *earlier* and *later* domains between PrEC and LNCaP.**

| | | |
|---|---|---|
| **Genes within ECDs** | **Variation, ESC** | DACT2, OR2AF1P, RAP2C, RAP2C-AS1, ROBO2P1, RP1-39J2.1, RP4-718N17.2 |
| | **Variation, no ESC** | AC012305.1, AC064874.1, AC092576.1, AGAP1, AGAP1-IT1, AP000472.2, BAMBI, C10orf126, CTD-2057J6.1, CTD-2057J6.2, GABRA3, GALNTL6, LINC00837, RNA5SP308, RNU4ATAC6P, RNU6-1067P, RNU6-270P, RNU6-764P, RP11-478H13.1, RP11-478H13.3, RP11-478H13.4, RP11-492M23.2, TPRKBP1 |
| | **No variation, ESC** | WAC, WAC-AS1 |
| | | |
| **Genes within LCDs** | **Variation, ESC** | AC008394.1, AC008694.2, AC008834.1, AC020594.5, AC021660.1, AC098847.1, AC105941.1, AC106801.1, ADAM19, ADAMTS6, ARSJ, ATP6V1G3, C12orf55, CH17-12M21.1, CPOX, CTB-109A12.1, CTB-47B11.1, CTB-47B11.3, CTD-2161E19.1, CTD-2215L10.1, CTSC, CWC27, CYFIP2, DCBLD2, EEF1A1P36, GAPDHP70, GLUD1P4, GRM5, GRM5-AS1, IL1RAPL1, KRT8P32, LINC01085, LTBP1, MIR181A1, MIR181A1HG, MIR181B1, MIR3166, MIR4280, MIR4636, MOXD1, MRPS35P2, MTRNR2L6, NEK7, NIPAL4, PDE4B, PDLIM1P4, PEBP1P3, PMAIP1, PTPRC, RAB38, RASA1, RASGRP3, RN7SKP34, RNA5SP91, RNA5SP92, RNU6-16P, RNU6-256P, RNU6-26P, RNU6-390P, RNU6-567P, RP11-1008C21.1, RP11-1008C21.2, RP11-104D3.1, RP11-104D3.2, RP11-143A12.3, RP11-164N3.1, RP11-164N3.2, RP11-164N3.3, RP11-16L9.1, RP11-16L9.2, RP11-16L9.3, RP11-16L9.4, RP11-227H4.1, RP11-26P13.2, RP11-274E7.1, RP11-274E7.2, RP11-307L14.1, RP11-307L14.2, RP11-382E9.1, RP11-397D21.1, RP11-474J18.1, RP11-474J18.2, RP11-553K8.2, RP11-553K8.5, RP11-669M16.2, RP11-72L22.1, RP11-824M15.3, RP11-866E20.1, RPS26P54, SEMA5A, SGIP1, SORL1, SOX30, ST3GAL6, ST3GAL6-AS1, STARD4-AS1, TRBV19, TRBV20-1, TRBV21-1, TRBV22-1, TRBV23-1, TRBV24-1, TRBV25-1, TRBVA, WWP1P1, Y_RNA |
| | **Variation, no ESC** | AC004543.2, AC005062.2, AC005083.1, AC010468.1, AC010468.3, AC023590.1, AC068610.3, AC068610.5, AC090673.2, AC099342.1, AIM2, AKAP2, AKT3, AKT3-IT1, AL137251.1, AL359753.1, AL512505.1, AP004372.1, ATG3, BTLA, C3orf52, C4orf51, C9orf152, CAMK4, CCDC50, CCDC80, CD1D, CD200, CD200R1L, CD5L, CDH13, COG6, CTA-293F17.1, CTC-551A13.1, CTC-551A13.2, DAZL, ELL2P1, ETS1, EXT1, FCRL1, FLI1, FLI1-AS1, FOXP2, FRMD4A, FRMPD4, GCSAM, GORAB, HMGA2, IFI16, IL1RAPL1, ITGB8, KCNJ1, KIRREL, KIRREL-IT1, L3MBTL3, LAMA2, LHFP, LINC00305, LINC00710, LLPH, MACC1, MACC1-AS1, MDFIC, MIR4305, MIR567, MMAA, MRPS21P2, NTM, NTM-IT, OPCML, OR7E100P, OSTN, OSTN-AS1, PALM2, PALM2-AKAP2, PRRX1, RAC1P6, RNA5SP362, RNU6-861P, RP1-186E20.1, RP1-251M9.2, RP1-79C4.1, RP1-79C4.4, RP11-1007G5.2, RP11-118B13.1, RP11-142M10.2, RP11-151F5.2, RP11-180K7.1, RP11-181K12.2, RP11-203B7.1, RP11-203B7.2, RP11-231E6.1, RP11-264E20.1, RP11-264E20.2, RP11-278H7.1, RP11-308K19.2, RP11-366L20.2, RP11-366L20.3, RP11-366L20.4, RP11-397C18.2, RP11-404O13.1, RP11-404O13.4, RP11-404O13.5, RP11-406O23.2, RP11-451O13.1, RP11-486P11.1, RP11-520H16.4, RP11-545I10.2, RP11-557J10.3, RP11-557J10.4, RP11-572C15.3, RP11-572C15.5, RP11-572C15.6, RP11-576I22.2, RP11-647P12.2, RP11-651K21.1, RP11-697E14.2, RP11-702B10.1, RP11-702B10.2, RP11-73O6.3, RP11-744N12.3, RP11-745O10.2, RP11-757F18.3, RP11-757F18.5, RP11-90K6.1, RPL12P39, RPL21P75, SAMD12, SAMD3, SERPINB8, SFTA1P, SLC35A5, SLC9C1, SNORD56, TMBIM4, TMEM200A, TNIP3, TSLP, UTS2B, WDR36, Y_RNA, ZNF827 |
| | **No variation, ESC** | RP11-669M16.1 |

**Supplementary Table 2: List of genes found in ECDs and LCDs.**

| Name | Forward (5' – 3') | Reverse (5' – 3') | Size (bp) |
|---|---|---|---|
| BMP1 | GATGAAGCCTCGACCCCTAGAT | ACCCGTCAGAGACGAACTTGAG | 177 |
| DPPA2 | AGGTGGACAGCGAAGACAGAAC | GGCCATCAGCAGTGTCCTAAAC | 168 |

**Supplementary Table 3: qRT-PCR primers of known early and late genes used for the validation of FACS sorting.**
Primers were obtained from Ryba *et al.* (2011)[7].

| Sample | DNA (ng) | | total reads | unaligned rate | multiple rate | clonal rate | unique reads |
|---|---|---|---|---|---|---|---|
| PrEC - G1 A | 33.75 | | 30,135,484 | 24% | 11% | 5% | 18,733,747 |
| PrEC - S2 A | 30.45 | | 29,586,038 | 25% | 7% | 3% | 19,422,895 |
| PrEC - S2 A | 39.00 | | 30,159,965 | 25% | 7% | 2% | 20,096,375 |
| PrEC - S3 A | 27.45 | | 28,516,781 | 25% | 8% | 3% | 18,697,557 |
| PrEC - S4 A | 28.05 | | 30,468,152 | 26% | 9% | 4% | 19,063,711 |
| PrEC - G2 A | 22.35 | | 30,337,296 | 27% | 11% | 7% | 17,714,661 |
| PrEC - G1 B | 18.00 | | 30,904,860 | 28% | 8% | 5% | 18,899,436 |
| PrEC - S2 B | 20.10 | | 27,847,360 | 25% | 7% | 3% | 18,607,367 |
| PrEC - S2 B | 25.80 | | 14,907,473 | 24% | 7% | 1% | 10,177,722 |
| PrEC - S3 B | 27.45 | | 31,971,081 | 35% | 7% | 3% | 18,053,236 |
| PrEC - S4 B | 22.05 | | 30,516,642 | 25% | 9% | 4% | 19,260,281 |
| PrEC - G2 B | 16.05 | | 30,173,914 | 27% | 11% | 5% | 17,980,647 |
| LNCaP - G1 A | 26.10 | | 28,326,880 | 26% | 8% | 4% | 17,940,873 |
| LNCaP - S2 A | 37.05 | | 28,053,807 | 25% | 7% | 2% | 18,559,668 |
| LNCaP - S2 A | 16.65 | | 27,559,871 | 27% | 7% | 3% | 17,629,910 |
| LNCaP - S3 A | 22.95 | | 29,053,372 | 26% | 7% | 3% | 18,884,690 |
| LNCaP - S4 A | 17.10 | | 30,758,466 | 27% | 9% | 5% | 18,941,203 |
| LNCaP - G2 A | 23.70 | | 32,087,371 | 29% | 11% | 6% | 18,136,642 |
| LNCaP - G1 B | 17.40 | | 15,960,930 | 26% | 9% | 4% | 10,027,200 |
| LNCaP - S2 B | 37.20 | | 28,238,523 | 27% | 7% | 4% | 17,885,113 |
| LNCaP - S2 B | 47.70 | | 26,673,785 | 25% | 7% | 3% | 17,657,594 |
| LNCaP - S3 B | 33.60 | | 30,303,383 | 24% | 7% | 3% | 20,053,111 |
| LNCaP - S4 B | 48.30 | | 28,333,690 | 27% | 9% | 5% | 17,195,794 |
| LNCaP - G2 B | 34.35 | | 28,148,820 | 27% | 13% | 8% | 15,695,571 |

**Supplementary Table 4: Sequencing metrics and QC for RepliSeq.**
PrEC and LNCaP were sorted into 6 fractions in duplicate. DNA (ng) refers to the amount of DNA sent for sequencing at the USC Epigenome Centre. 'Total reads' refers to the number of raw reads obtained for each sample; 'unique reads' refers to the final numbers that were suitable for further analyses.

| Cell line | Data Type | ENCODE link | Comments |
|---|---|---|---|
| HMEC | ChIP-seq H3K4me3 | https://www.encodeproject.org/experiments/ENCSR807YQE/ | |
| HMEC | ChIP-seq H3K4me1 | https://www.encodeproject.org/experiments/ENCSR521FND/ | |
| HMEC | ChIP-seq H3K36me3 | https://www.encodeproject.org/experiments/ENCSR597RXT/ | |
| HMEC | ChIP-seq DNAse1 HS | https://www.encodeproject.org/experiments/ENCSR000ENV/ | |
| HMEC | ChIP-seq H3K27me3 | https://www.encodeproject.org/experiments/ENCSR134HVI/ | |
| HMEC | ChIP-seq H3K9me3 | https://www.encodeproject.org/experiments/ENCSR668GKQ/ | |
| MCF7 | ChIP-seq H3K4me3 | https://www.encodeproject.org/experiments/ENCSR985MIB/ | Pooled replicate peaks |
| MCF7 | ChIP-seq H3K4me1 | https://www.encodeproject.org/experiments/ENCSR493NBY/ | Pooled replicate peaks |
| MCF7 | ChIP-seq H3K36me3 | https://www.encodeproject.org/experiments/ENCSR610IYQ/ | Downloaded fastq from ENCODE and processed in-house |
| MCF7 | ChIP-seq DNAse1 HS | https://www.encodeproject.org/experiments/ENCSR000EPH/ | Pooled replicate peaks |
| MCF7 | ChIP-seq H3K27me3 | https://www.encodeproject.org/experiments/ENCSR761DLU/ | Pooled replicate peaks |
| MCF7 | ChIP-seq H3K9me3 | https://www.encodeproject.org/experiments/ENCSR999WHE/ | Pooled replicate peaks |
| HELA S3 | polyA RNA-seq | https://www.encodeproject.org/experiments/ENCSR000CPR/ | Lab: Thomas Gingeras, CSHL |
| K562 | polyA RNA-seq | https://www.encodeproject.org/experiments/ENCSR000CPH/ | Lab: Thomas Gingeras, CSHL |
| SK-N-SH | polyA RNA-seq | https://www.encodeproject.org/experiments/ENCSR000CTT/ | Lab: Thomas Gingeras, CSHL |
| HepG2 | polyA RNA-seq | https://www.encodeproject.org/experiments/ENCSR000CPE/ | Lab: Thomas Gingeras, CSHL |
| MCF7 | polyA RNA-seq | https://www.encodeproject.org/experiments/ENCSR000CPT/ | Lab: Thomas Gingeras, CSHL |
| IMR90 | polyA RNA-seq | https://www.encodeproject.org/experiments/ENCSR000CTQ/ | Lab: Thomas Gingeras, CSHL |
| BJ | polyA RNA-seq | https://www.encodeproject.org/experiments/ENCSR000COP/ | Lab: Thomas Gingeras, CSHL |
| NHEK | polyA RNA-seq | https://www.encodeproject.org/experiments/ENCSR000CPL/ | Lab: Thomas Gingeras, CSHL |
| HUVEC | polyA RNA-seq | https://www.encodeproject.org/experiments/ENCSR000COZ/ | Lab: Thomas Gingeras, CSHL |
| GM12878 | polyA RNA-seq | https://www.encodeproject.org/experiments/ENCSR000COQ/ | Lab: Thomas Gingeras, CSHL |
| H1 ESC | polyA RNA-seq | https://www.encodeproject.org/experiments/ENCSR000COU/ | Lab: Thomas Gingeras, CSHL |

**Supplementary Table 5: Public ENCODE datasets used in this study.**

| Data Type | Cell Line | Source | Accession |
|---|---|---|---|
| Repli-Seq | PrEC | This publication | GSE98732 |
| Repli-Seq | LNCaP | This publication | GSE98732 |
| H3K4me3 ChIP-seq | PrEC | Bert et. al., Cancer Cell 2013 | GSE38685 |
| H3K4me3 ChIP-seq | LNCaP | Bert et. al., Cancer Cell 2013 | GSE38685 |
| H3K4me1 ChIP-seq | PrEC | Taberlay et. al., Genome Research 2014 | GSE57498 |
| H3K4me1 ChIP-seq | LNCaP | Taberlay & Achinger-Kawecka et. al., Genome Research 2016 | GSE73785 |
| H3K36me3 ChIP-seq | PrEC | This publication | GSE98732 |
| H3K36me3 ChIP-seq | LNCaP | This publication | GSE98732 |
| H3K27ac ChIP-seq | PrEC | Taberlay et. al., Genome Research 2014 | GSE57498 |
| H3K27ac ChIP-seq | LNCaP | Taberlay & Achinger-Kawecka et. al., Genome Research 2016 | GSE73785 |
| H2AZac ChIP-seq | PrEC | Valdes-Mora et. al., Nature Comms. 2017 | GSE76337 |
| H2AZac ChIP-seq | LNCaP | Valdes-Mora et. al., Nature Comms. 2017 | GSE76337 |
| H3K27me3 ChIP-seq | PrEC | Bert et. al., Cancer Cell 2013 | GSE38685 |
| H3K27me3 ChIP-seq | LNCaP | Bert et. al., Cancer Cell 2013 | GSE38685 |
| H3K9me3 ChIP-seq | PrEC | This publication | GSE98732 |
| H3K9me3 ChIP-seq | LNCaP | This publication | GSE98732 |
| Lamin A/C ChIP-seq | PrEC | This publication | GSE98732 |
| Lamin A/C ChIP-seq | LNCaP | This publication | GSE98732 |
| Lamin B1 ChIP-seq | PrEC | This publication | GSE98732 |
| Lamin B1 ChIP-seq | LNCaP | This publication | GSE98732 |
| WGBS | PrEC | Pidsley & Zotenko et. al., Genome Biology 2016 | GSE86833 |
| WGBS | LNCaP | Pidsley & Zotenko et. al., Genome Biology 2016 | GSE86833 |
| WGBS | MCF7 | This publication | GSE98732 |
| RNA-Seq | PrEC | Taberlay & Achinger-Kawecka et. al., Genome Research 2016 | GSE73785 |
| RNA-Seq | LNCaP | Taberlay & Achinger-Kawecka et. al., Genome Research 2016 | GSE73785 |
| DNase1 HS | PrEC | This publication | GSE98732 |
| DNase1 HS | LNCaP | This publication | GSE98732 |

**Supplementary Table 6: A summary of datasets generated and used in this study.**

## Supplementary Methods

### WGBS data processing

Adaptor sequences and poor quality bases were removed using Trim Galore (v0.2.8, http://www.bioinformatics.babraham.ac.uk/projects/trim_galore/) in paired-end mode with default parameters. bwa-meth[8] (v0.10) was used to align reads to hg19 using default parameters. PCR duplicates were removed using Picard (v1.91, http://broadinstitute.github.io/picard). Count tables of the number of methylated and unmethylated bases sequenced at each CpG site in the genome were constructed using the 'tabulate' module of bwa-meth and BisSNP[9] (v0.82.2) with default parameters.

### RNA-seq data processing

Paired-end reads (100 bp) in biological triplicate for PrEC and LNCaP were processed as previously described[10] (See Supplementary Methods). using Trim Galore (v0.4.0, parameter settings: --fastqc --paired --retain_unpaired --length 16) and STAR[11] (version 2.4.0j, parameter settings: --quantMode TranscriptomeSAM --outFilterMatchNmin 101) for mapping reads to the hg19 human transcriptome build (GENCODE 19[12]). For expression comparison between PrEC and LNCaP, mapped reads where counted into genes using featureCounts[13] (v1.4.6-p4) and differential analysis was performed using edgeR[14]. RNA-seq processed for PCA and hierarchical clustering were performed with updated program versions, Trim Galore (v0.4.5_dev) and STAR (v2.5.4b), and mapped reads were counted into genes using RSEM[15] (v1.3.0). Datasets were normalised using ERCC controls before calculating logCPMs (edgeR[14]).

## Supplementary References


1 Hyrien, O. Peaks cloaked in the mist: the landscape of mammalian replication origins. *The Journal of cell biology* **208**, 147-160, (2015).
2 Petryk, N. *et al.* Replication landscape of the human genome. *Nat Commun* **7**, 10208, (2016).
3 Brinkman, A. B. *et al.* Partially methylated domains are hypervariable in breast cancer and fuel widespread CpG island hypermethylation. *Preprint at biorxiv* https://doi.org/10.1101/305193, (2018).
4 Berman, B. P. *et al.* Regions of focal DNA hypermethylation and long-range hypomethylation in colorectal cancer coincide with nuclear lamina-associated domains. *Nature genetics* **44**, 40-46, (2012).
5 Sproul, D. *et al.* Tissue of origin determines cancer-associated CpG island promoter hypermethylation patterns. *Genome Biol* **13**, R84, (2012).
6 Robinson, D. *et al.* Integrative clinical genomics of advanced prostate cancer. *Cell* **161**, 1215-1228, (2015).
7 Ryba, T., Battaglia, D., Pope, B. D., Hiratani, I. & Gilbert, D. M. Genome-scale analysis of replication timing: from bench to bioinformatics. *Nature protocols* **6**, 870-895, (2011).
8 Pedersen, B. S., Eyring, K., De, S., Yang, I. V. & Schwartz, D. A. Fast and accurate alignment of long bisulfite-seq reads. *Preprint at arxiv* https://arxiv.org/abs/1401.1129, (2014).
9 Liu, Y., Siegmund, K. D., Laird, P. W. & Berman, B. P. Bis-SNP: Combined DNA methylation and SNP calling for Bisulfite-seq data. *Genome biology* **13**, R61, (2012).
10 Taberlay, P. C. *et al.* Three-dimensional disorganization of the cancer genome occurs coincident with long-range genetic and epigenetic alterations. *Genome research* **26**, 719-731, (2016).

11 Dobin, A. *et al.* STAR: ultrafast universal RNA-seq aligner. *Bioinformatics* **29**, 15-21, (2013).
12 Harrow, J. *et al.* GENCODE: the reference human genome annotation for The ENCODE Project. *Genome research* **22**, 1760-1774, (2012).
13 Liao, Y., Smyth, G. K. & Shi, W. featureCounts: an efficient general purpose program for assigning sequence reads to genomic features. *Bioinformatics* **30**, 923-930, (2013).
14 Robinson, M. D., McCarthy, D. J. & Smyth, G. K. edgeR: a Bioconductor package for differential expression analysis of digital gene expression data. *Bioinformatics* **26**, 139-140, (2010).
15 Li, B. & Dewey, C. N. RSEM: accurate transcript quantification from RNA-Seq data with or without a reference genome. *BMC Bioinformatics* **12**, 323, (2011).
