# Supplementary material for: Replication timing and epigenome remodelling are associated with the nature of chromosomal rearrangements in cancer
Source: Nat Commun. 2019 Jan 24;10:416. doi: 10.1038/s41467-019-08302-1 (PMC6345877; doi:10.1038/s41467-019-08302-1)
Supplement: Supplementary file 2 — Description of Additional Supplementary Files [file 41467_2019_8302_MOESM2_ESM.pdf]

Description of Additional Supplementary Files

File Name: Supplementary Data 1
Description: **Prostate cancer gene fusions and replication timing.** A table of prostate cancer gene fusions documented in Robinson et al. (2015), with their corresponding replication timing values in PrEC and LNCaP. Table columns from left to right are as follows: 5' breakpoint chromosome; 5' breakpoint coordinate; 5' gene name; PrEC replication timing (WA) at 5' breakpoint; LNCaP replication timing (WA) at 5' breakpoint; difference in timing between PrEC and LNCaP at the 5' breakpoint; 3' breakpoint chromosome; 3' breakpoint coordinate; 3' gene name; PrEC replication timing (WA) at 3' breakpoint; LNCaP replication timing (WA) at 3' breakpoint; difference in timing between PrEC and LNCaP at the 3' breakpoint; difference in PrEC timing between 5' and 3' breakpoints; difference in LNCaP timing between 5' and 3' breakpoints.
