# Supplementary material for: Replication timing and epigenome remodelling are associated with the nature of chromosomal rearrangements in cancer
Source: Nat Commun. 2019 Jan 24;10:416. doi: 10.1038/s41467-019-08302-1 (PMC6345877; doi:10.1038/s41467-019-08302-1)
Supplement: Supplementary file 4 — Reporting Summary [file 41467_2019_8302_MOESM4_ESM.pdf]

nature research

Corresponding author(s): Susan J Clark

Reporting Summary

Nature Research wishes to improve the reproducibility of the work that we publish. This form provides structure for consistency and transparency in reporting. For further information on Nature Research policies, see Authors & Referees and the Editorial Policy Checklist.

Statistical parameters

When statistical analyses are reported, confirm that the following items are present in the relevant location (e.g. figure legend, table legend, main text, or Methods section).

n/a	Confirmed	
☐	☒	The exact sample size (*n*) for each experimental group/condition, given as a discrete number and unit of measurement
☒	☐	An indication of whether measurements were taken from distinct samples or whether the same sample was measured repeatedly
☐	☒	The statistical test(s) used AND whether they are one- or two-sided<br>*Only common tests should be described solely by name; describe more complex techniques in the Methods section.*
☒	☐	A description of all covariates tested
☐	☒	A description of any assumptions or corrections, such as tests of normality and adjustment for multiple comparisons
☐	☒	A full description of the statistics including central tendency (e.g. means) or other basic estimates (e.g. regression coefficient) AND variation (e.g. standard deviation) or associated estimates of uncertainty (e.g. confidence intervals)
☐	☒	For null hypothesis testing, the test statistic (e.g. *F*, *t*, *r*) with confidence intervals, effect sizes, degrees of freedom and *P* value noted<br>*Give P values as exact values whenever suitable.*
☒	☐	For Bayesian analysis, information on the choice of priors and Markov chain Monte Carlo settings
☒	☐	For hierarchical and complex designs, identification of the appropriate level for tests and full reporting of outcomes
☒	☐	Estimates of effect sizes (e.g. Cohen's *d*, Pearson's *r*), indicating how they were calculated
☐	☒	Clearly defined error bars<br>*State explicitly what error bars represent (e.g. SD, SE, CI)*

Our web collection on statistics for biologists may be useful.

Software and code

Policy information about availability of computer code

Data collection	Repli-Seq<br>- Bowtie v1.1.0<br><br>WGBS<br>- Trim Galore v0.2.8<br>- Bwa-meth v0.10<br>- Picard v1.91<br>- BisSNP v0.82.2<br>- MethPipe v3.4.2<br><br>RNA-seq PrEC vs. LNCaP<br>- Trim Galore v0.4.0<br>- STAR v2.4.0j<br>- Subread suite v1.4.6-p4<br><br>RNA-seq PCA and hclust<br>- Trim Galore v0.4.5_dev<br>- STAR v2.5.4b<br>- RSEM v1.3.0

ChIP-seq
- Bowtie v1.1.0
- Peak Ranger v1.16
- EDD (https://github.com/CollasLab/edd)
- F-seq v1.84
- NGSANE v0.5.2.0 (https://github.com/BauerLab/ngsane)

Data analysis

All analysis was performed using open source software.

R software and packages: R v3.2.3, GenomicRanges v1.22.4, GenomicAlignments v1.6.3, rtracklayer v1.30.4, edgeR v3.12.1 limma v3.26.9, LOLA v1.0.0, Repitools v1.16.0, aaRon v0.9.5 (https://github.com/astatham/aaRon), GenomicFeatures v1.22.13, genomation v1.2.2, BSgenome.Hsapiens.UCSC.hg19 v1.4.0, data.table v1.9.6, fpc v2.1-11, preprocessCore v1.32.0, ggthemes v3.0.3, reshape2 v1.4.1, ggplot2 v2.2.1, seqplots v1.8.1

Other software: chromHMM v1.10, IGV v2.3.32

For manuscripts utilizing custom algorithms or software that are central to the research but not yet described in published literature, software must be made available to editors/reviewers upon request. We strongly encourage code deposition in a community repository (e.g. GitHub). See the Nature Research guidelines for submitting code & software for further information.

Data

Policy information about availability of data

All manuscripts must include a data availability statement. This statement should provide the following information, where applicable:
- Accession codes, unique identifiers, or web links for publicly available datasets
- A list of figures that have associated raw data
- A description of any restrictions on data availability

Raw and processed Repli-Seq and ChIP-seq data from this study have been submitted to the NCBI Gene Expression Omnibus (GEO; http://www.ncbi.nlm.nih.gov/geo/) under accession number GSE98732. RNA-seq data is available under GEO accession number GSE73784. ChIP-seq data from Valdes-Mora et al., Bert et al., Taberlay et al. and Taberlay et al. are available under GEO accession numbers GSE76337, GSE38685, GSE57498, GSE73785, respectively. WGBS data from Pidsley et al. are available under GEO accession number GSE86833. Prostate cancer patient WGBS are available under GEO accession number GSE104789. LRES and LREA domains are from Coolen et al. and Bert et al.

Field-specific reporting

Please select the best fit for your research. If you are not sure, read the appropriate sections before making your selection.

☒ Life sciences ☐ Behavioural & social sciences ☐ Ecological, evolutionary & environmental sciences

For a reference copy of the document with all sections, see nature.com/authors/policies/ReportingSummary-flat.pdf

Life sciences study design

All studies must disclose on these points even when the disclosure is negative.

Sample size	No human or animal subjects necessitating sample size calculations were used in this study.
Data exclusions	No data was excluded from analysis.
Replication	For PrEC and LNCaP, Repli-Seq was performed in duplicate and RNA-seq was performed in triplicate. WGBS observations were replicated in the prostate cancer cell model, the breast cancer cell model and public patient breast and prostate cancer data. ChIP-seq observations were replicated in the prostate and breast cancer cell model systems where public data was available.
Randomization	No randomisation was required in our study as no human or animal subjects were used.
Blinding	No blinding was required in our study as no human or animal subjects were used.

Reporting for specific materials, systems and methods

Materials & experimental systems

n/a	Involved in the study	
☒	☐	Unique biological materials
☐	☒	Antibodies
☐	☒	Eukaryotic cell lines
☒	☐	Palaeontology
☒	☐	Animals and other organisms
☒	☐	Human research participants

Methods

n/a	Involved in the study	
☐	☒	ChIP-seq
☒	☐	Flow cytometry
☒	☐	MRI-based neuroimaging

Antibodies

Antibodies used	Antibodies used were H3K4me3 (Abcam, #ab8580), H3K4me1 (Active Motif, #39297), H3K36me3 (Abcam, #ab9050), H3K27ac (Active Motif, #39133), H2AZac (Abcam, #ab18262), H3K27me3 (Millipore, #07-449), H3K9me3 (Diagenode, #C15500003), Lamin A/C (Santa Cruz, #sc7292), Lamin B1 (Abcam, #ab16048) and anti-BrdU (BD Pharmingen, #555627).
Validation	The following antibodies are validated for ChIP-seq in the Antibody Validation Database (Egelhofer et al. (2010)): H3K4me3 (Abcam, #ab8580), H3K4me1 (Active Motif, #39297), H3K36me3 (Abcam, #ab9050), H3K27ac (Active Motif, #39133) and H3K27me3 (Millipore, #07-449). H3K9me3 (Diagenode, #C15500003) is verified in Hattori et al. (2013). H2AZac (Abcam, #ab18262) is verified in Valdes-Mora et al. (2017). Lamin A/C (Santa Cruz, #sc7292) and Lamin B1 (Abcam, #ab16048) are verified in Lund et al. (2015). anti-BrdU (BD Pharmingen, #555627) is verified in Ryba et al. (2011).

Eukaryotic cell lines

Policy information about cell lines

Cell line source(s)	LNCaP prostate cancer cells (ATCC #CRL-1740). PrEC normal prostate epithelial cells (Cambrex Bio Science Cat. No. CC-2555: PrEC1 tissue acquisition no. #13683). MCF7 breast cancer cells (ATCC #HTB-22).
Authentication	All cell lines were authenticated by short-tandem repeat profiling (CellBank Australia, Westmead, NSW, Australia) and cultured for <6 months after authentication.
Mycoplasma contamination	All cell lines used in-house tested negative for mycoplasma using the MycoAlert Mycoplasma Detection Kit (Lonza, #LT07-318).
Commonly misidentified lines (See ICLAC register)	No cell lines from the ICLAC register were used.

ChIP-seq

Data deposition

☒ Confirm that both raw and final processed data have been deposited in a public database such as GEO.

☒ Confirm that you have deposited or provided access to graph files (e.g. BED files) for the called peaks.

Data access links *May remain private before publication.*	GSE98732
Files in database submission	GSM2610541 PrEC DNase-seq<br>GSM2610542 PrEC H3K36me3 ChIP-seq<br>GSM2610543 PrEC H3K9me3 ChIP-seq<br>GSM2610544 PrEC Lamin A/C ChIP-seq<br>GSM2610545 PrEC Lamin B1 ChIP-seq<br>GSM2610546 LNCaP DNase-seq<br>GSM2610547 LNCaP H3K36me3 ChIP-seq<br>GSM2610548 LNCaP H3K9me3 ChIP-seq<br>GSM2610549 LNCaP Lamin A/C ChIP-seq<br>GSM2610550 LNCaP Lamin B1 ChIP-seq
Genome browser session (e.g. UCSC)	no longer applicable

Methodology

Replicates	PrEC and LNCaP ChIP-seq results were validated with public ChIP-seq datasets where public data was available.
Sequencing depth	Cell Line ChIP Total Reads Unique Length<br>LNCaP H3K36me3 22,078,675 17,569,542 50bp SR<br>PrEC H3K36me3 26,782,127 21,571,988 50bp SR

	LNCaP H3K9me3 86,042,841 66,878,855 50bp SR<br>PrEC H3K9me3 96,443,494 72,162,479 50bp SR<br>PrEC LaminB1 47,545,622 37,839,707 50bp SR<br>LNCaP LaminB1 64,554,568 52,920,759 50bp SR<br>PrEC LaminA/C 46,405,109 37,827,185 50bp SR<br>LNCaP LaminA/C 50,790,631 40,252,089 50bp SR
Antibodies	Antibodies used were H3K27me3 (Millipore, #07-449), H3K9me3 (Diagenode, #C15500003), Lamin A/C (Santa Cruz, #sc7292), Lamin B1 (Abcam, #ab16048).
Peak calling parameters	Peaks were called with Peak Ranger version 1.16 using default parameters. Broad domains were called with Enriched Domain Detector (https://github.com/CollasLab/edd) using default parameters.
Data quality	ChIP-seq libraries were validated by qPCR (n=3). All peaks are below the Peak Ranger FDR cut off.
Software	ChIP-seq reads were aligned to hg19 using bowtie v1.1.0 allowing up to 3 mismatches, discarding ambiguous and clonal reads.
